# Supplementary figures and images for: Image-based analysis of living mammalian cells using label-free 3D refractive index maps reveals new organelle dynamics and dry mass flux
Source: PLoS Biol. 2019 Dec 19;17(12):e3000553. doi: 10.1371/journal.pbio.3000553 (PMC6922317; doi:10.1371/journal.pbio.3000553)

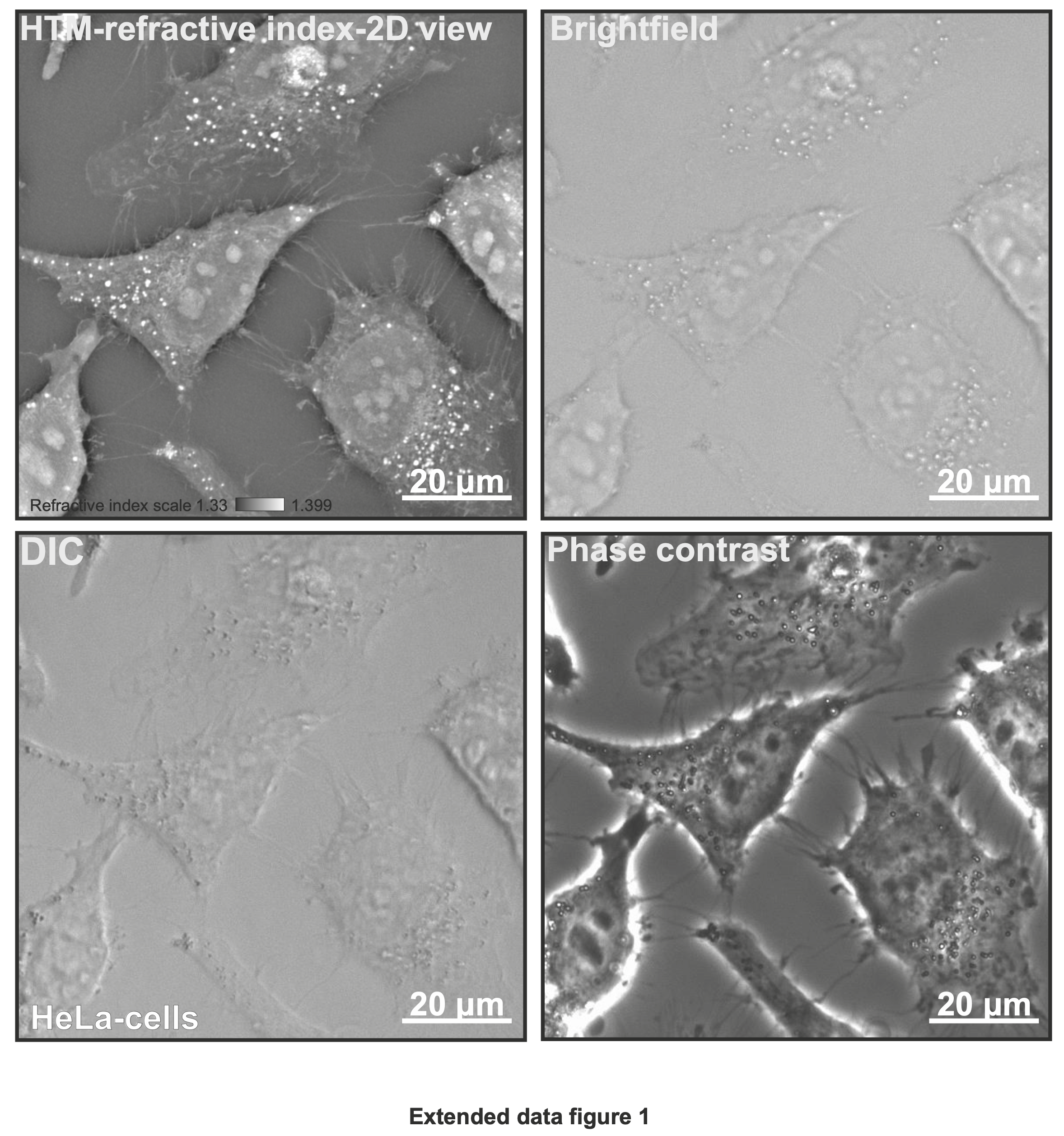

Supplement: S1 Fig — Brightfield, DIC, and phase contrast images show limited resolution and major artifacts. On the contrary, HTM images show low background and resolution high enough to observe details such as filopodia and mitochondria. DIC, differential interference contrast; HTM, holo-tomographic microscopy. (TIFF) [file pbio.3000553.s001.tiff]

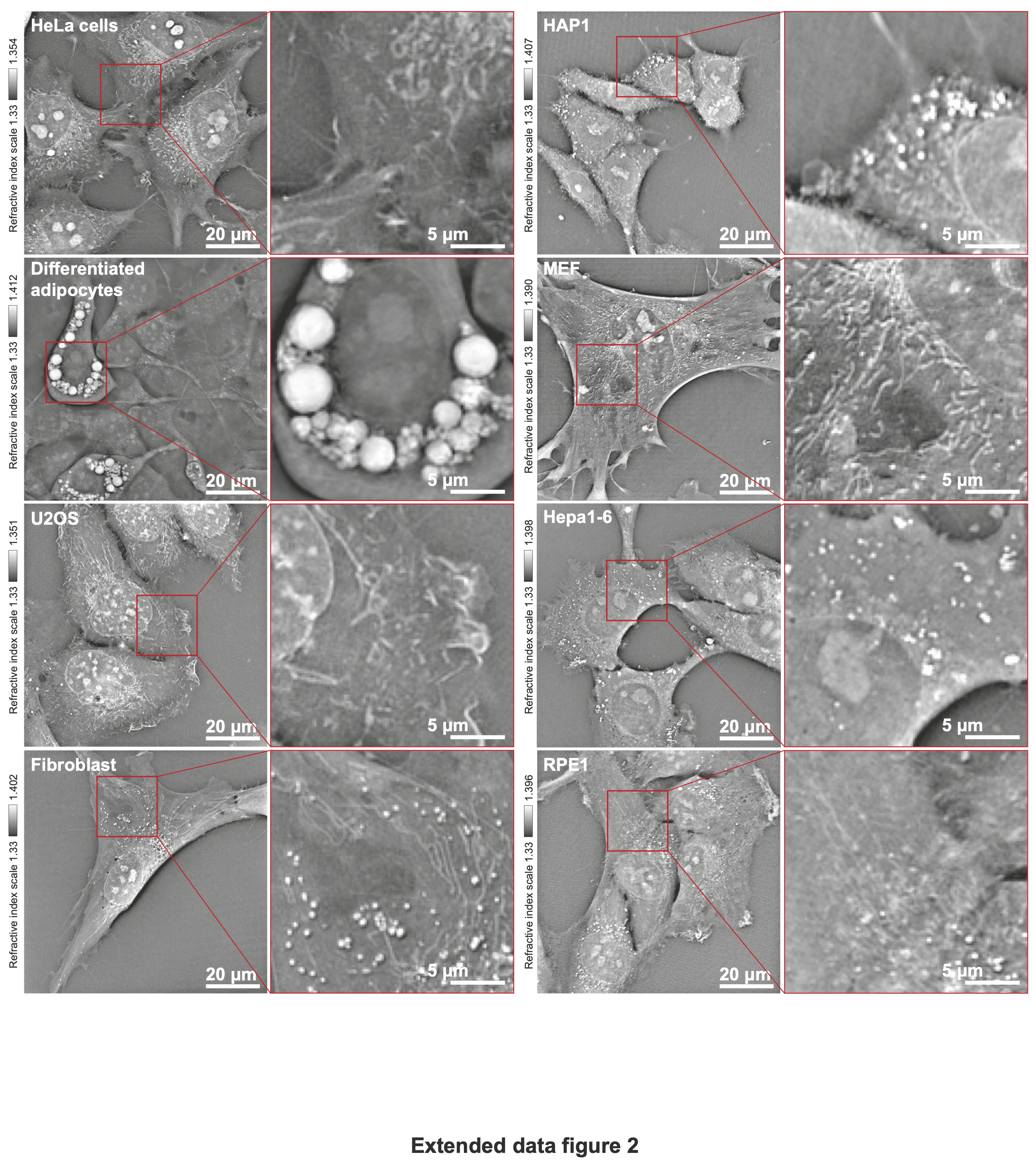

Supplement: S2 Fig — A wide range of cell lines are perfectly observable with holotomography. HTM, holo-tomographic microscopy. (TIFF) [file pbio.3000553.s002.tiff]

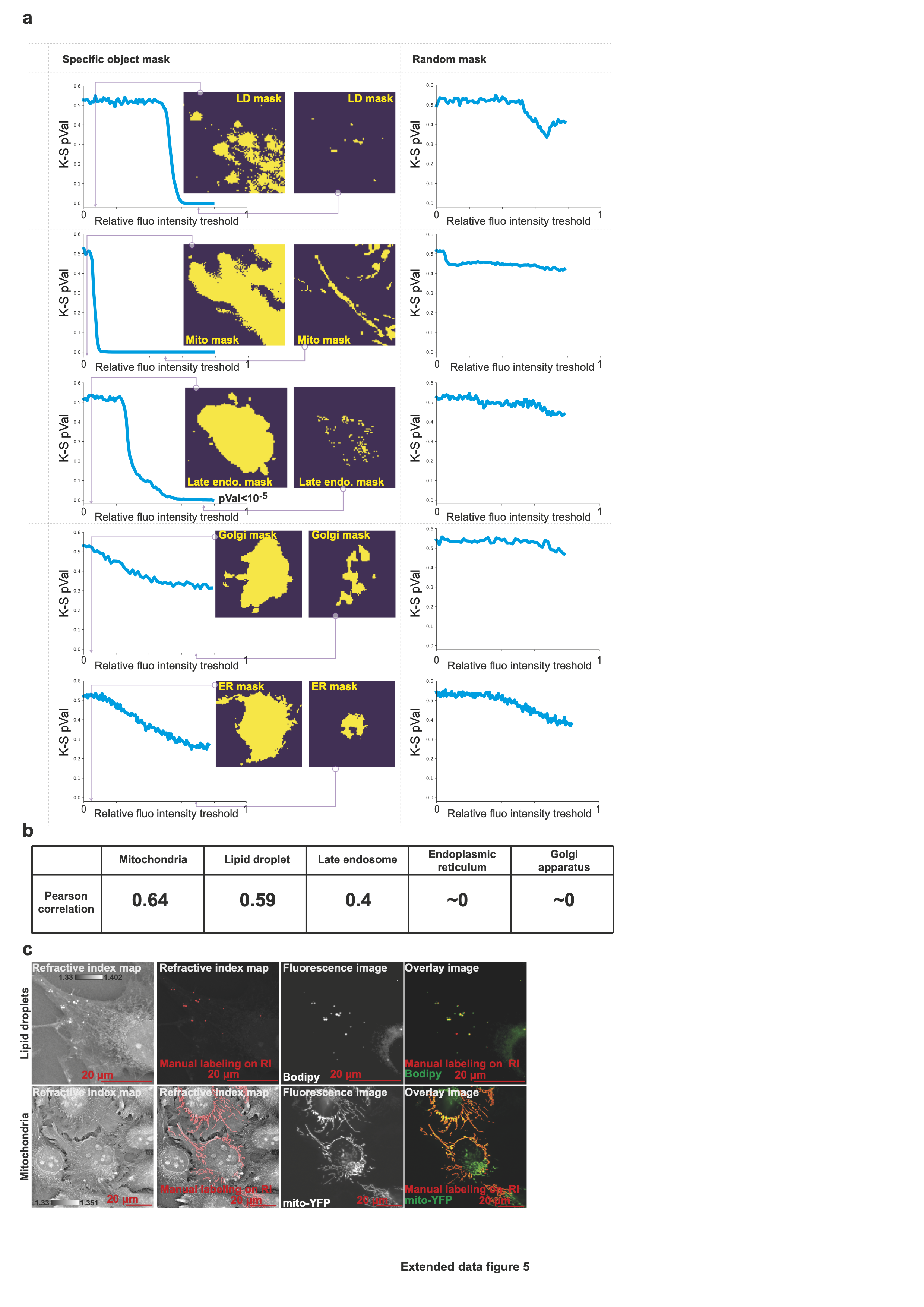

Supplement: S3 Fig — Methanol fixation affect organelles’ structural integrity; PFA is a viable solution for observing fixed cells with HTM. HTM, holo-tomographic microscopy; PFA, paraformaldehyde. (TIFF) [file pbio.3000553.s003.tiff]

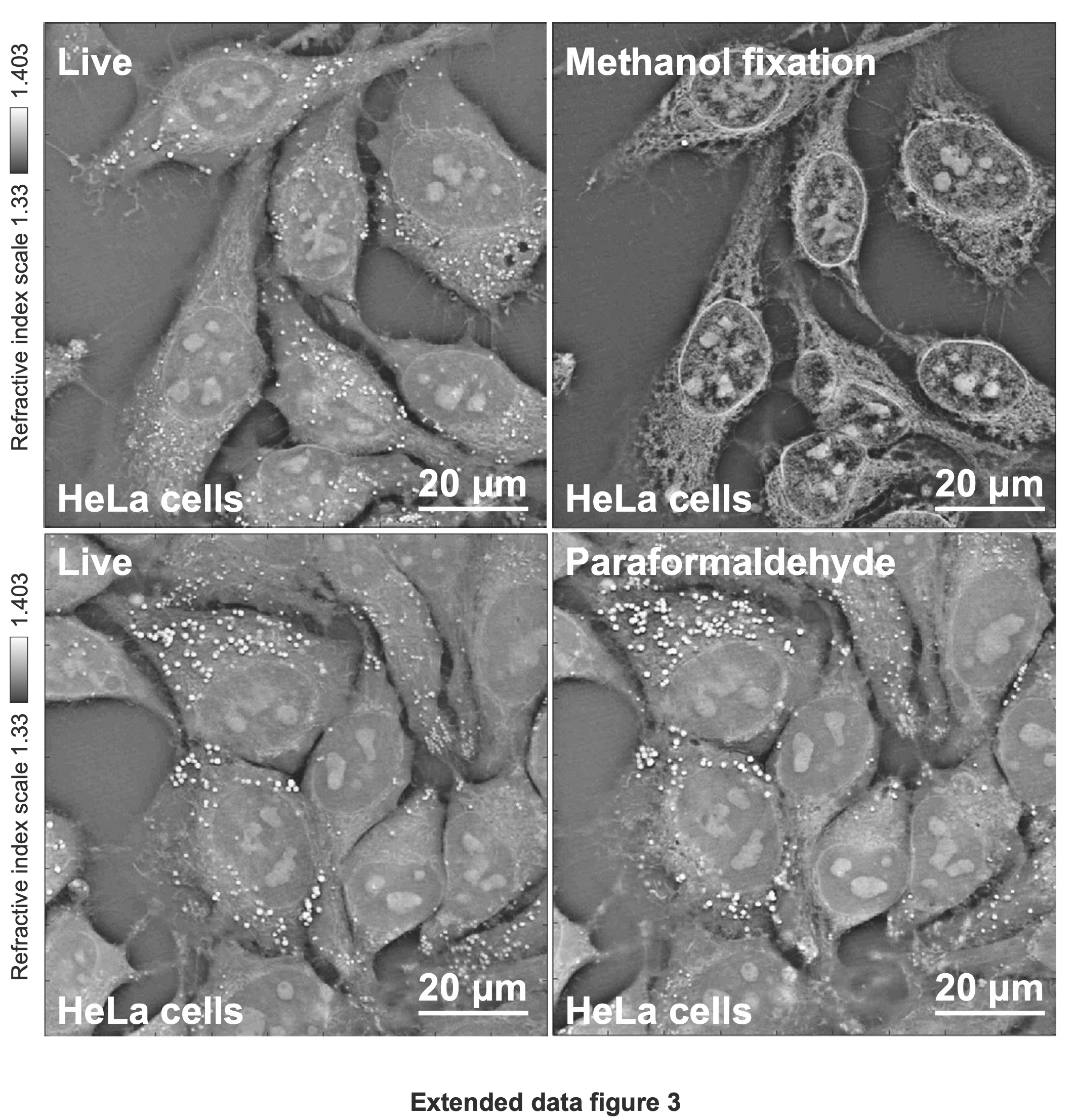

Supplement: S4 Fig — Visual comparison of HeLa cells’ RI map to (a) Golgi apparatus fluorescent signal (NAGTI-GFP), (b) an ER fluorescent signal (KDEL-GFP), or (c) a late endosome accumulation signal (Filipin). ER, endoplasmic reticulum; GFP, green fluorescent protein; KDEL, XXX; NAGTI, N-acetylglucosaminyltransferase I; RI, refractive index. (TIFF) [file pbio.3000553.s004.tiff]

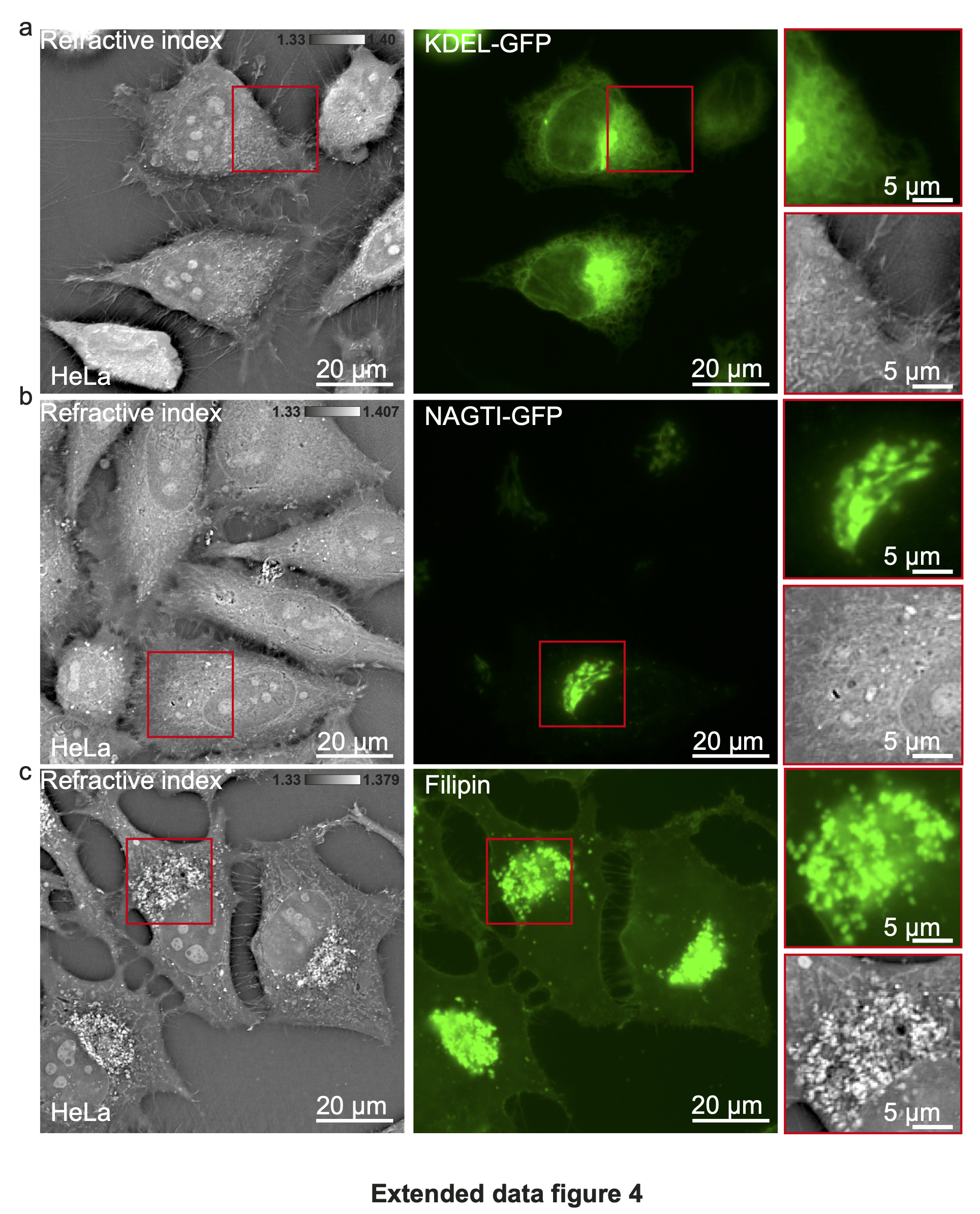

Supplement: S5 Fig — (a) Bootstrapped Kolmogorov–Smirnov test of the distribution of RI values under specific fluorescent signals against the global cellular RI distribution. Statistical test p-values are plotted as a function of the stringency of the threshold used to define the fluorescence mask from a specific or random fluorescent object signal. (b) Pearson correlation of various fluorescent and RI signals. (c) Comparison of Mito-YFP and Bodipy fluorescent signals with independent human expert labeling of mitochondria and LDs in RI maps. LD, lipid droplet; RI, refractive index; YFP, yellow fluorescent protein. (TIFF) [file pbio.3000553.s005.tiff]

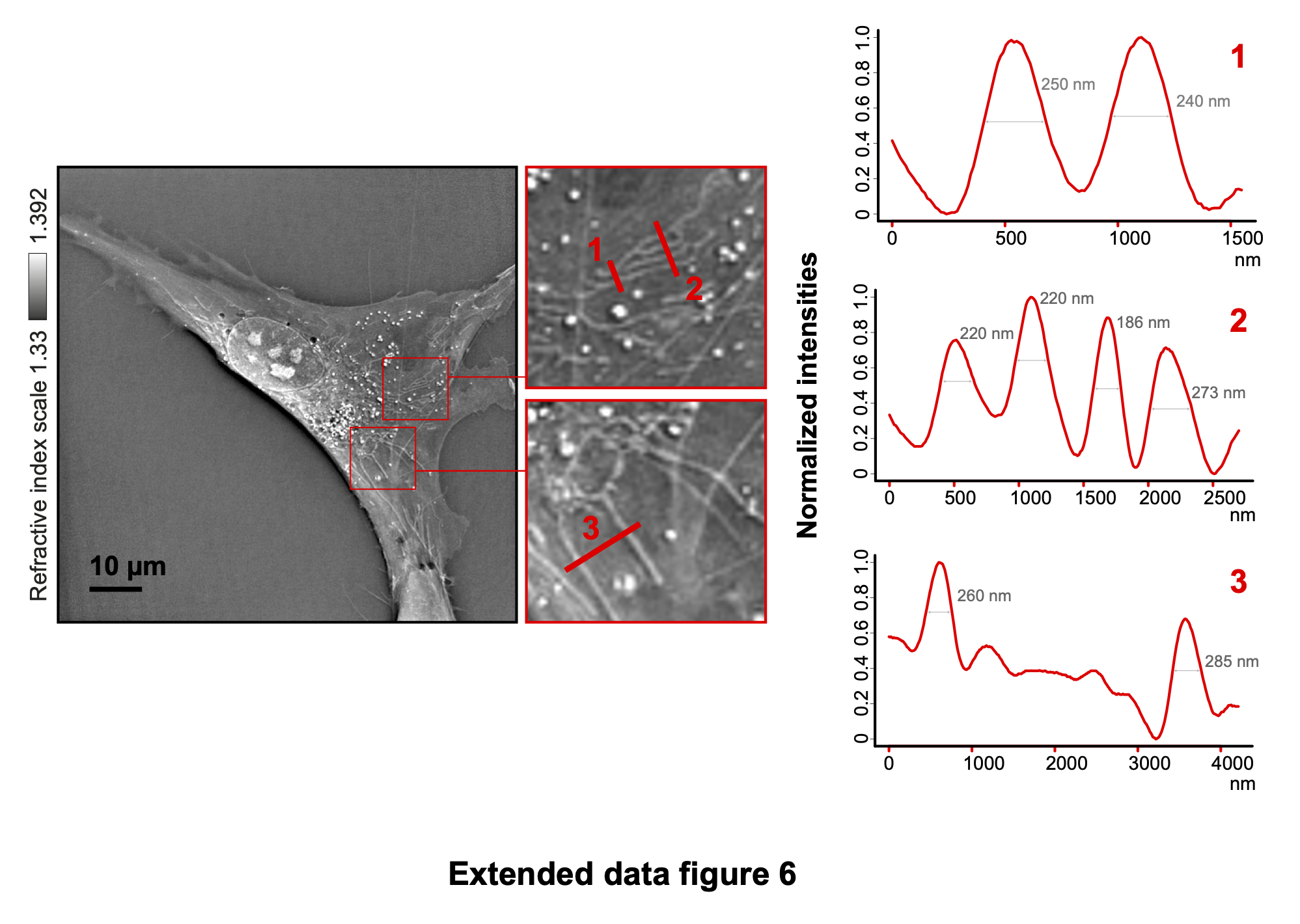

Supplement: S6 Fig — The mitochondrial thickness has been measured within fibroblasts as the width observed at the half maximum of the mitochondrial signal distribution. The signal distributions along transversal lines have been defined within the FIJI software on 0 padded image and are represented in the red enlargement squares (red lines 1–3). (TIFF) [file pbio.3000553.s006.tiff]

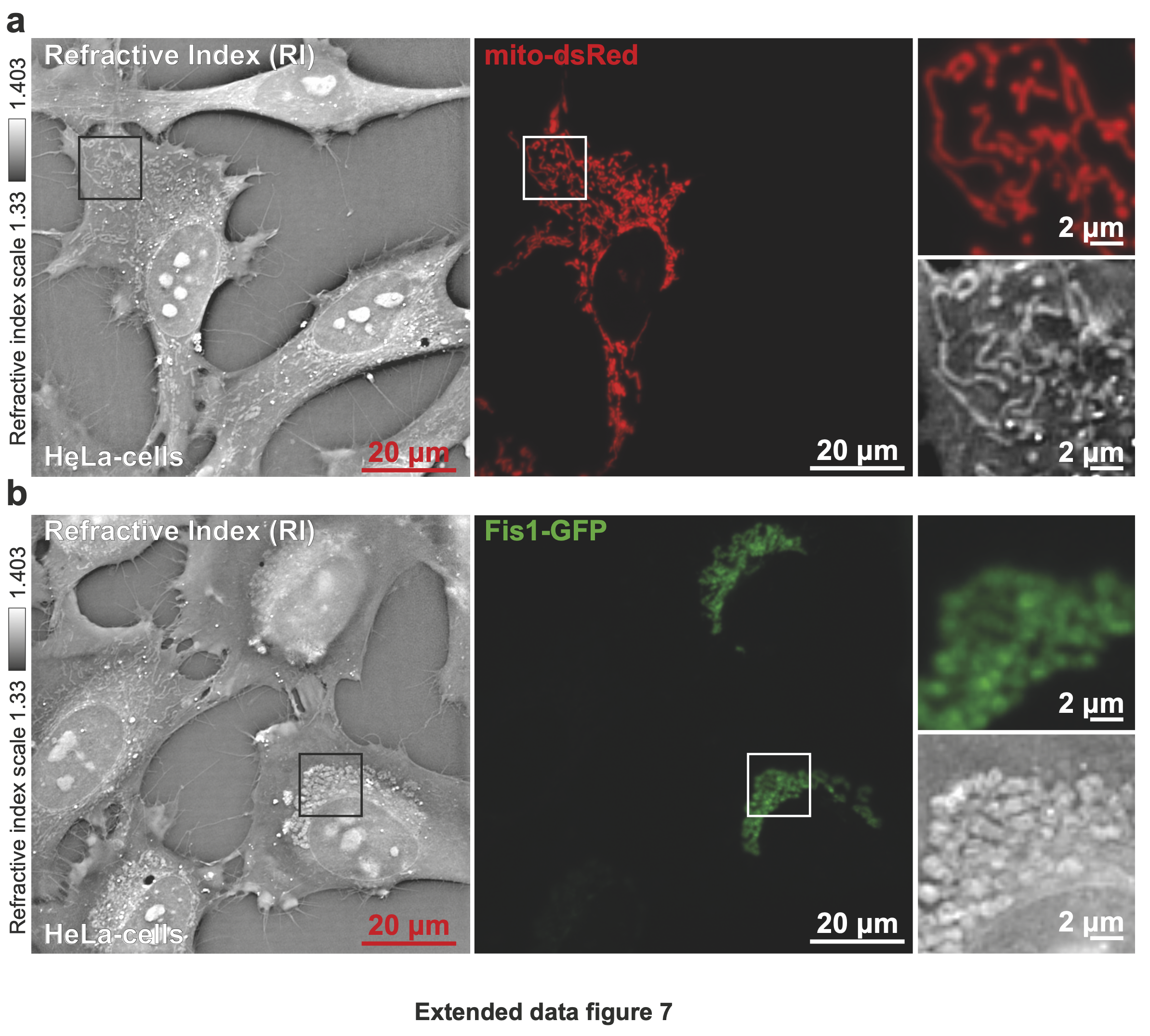

Supplement: S7 Fig — RI map of HeLa cells after transfection with (a) a neutral mitochondrial DsRed marker or (b) with the mitochondrial fusion protein Fis1-GFP. Ds, XXX; Fis1, fission protein 1; GFP, green fluorescent protein; RI, refractive index. (TIFF) [file pbio.3000553.s007.tiff]

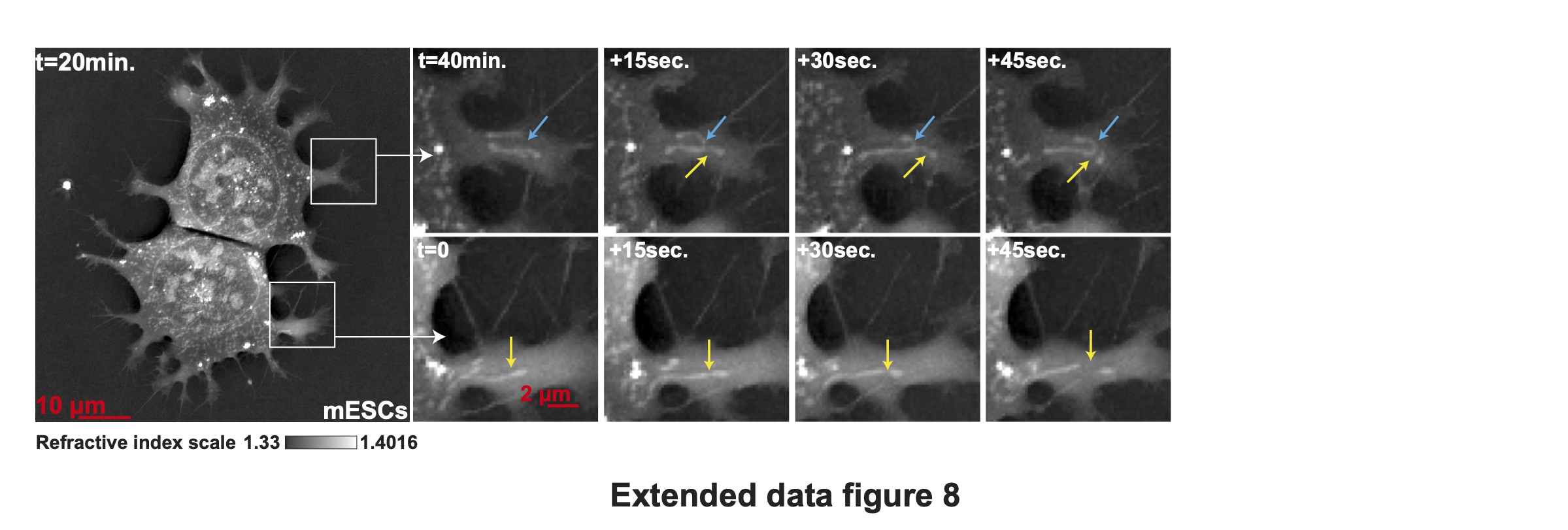

Supplement: S8 Fig — RI map of mESC times series showing mitochondrial fusion (blue arrow) and fission (yellow arrow) events. (related to S1 Movie). HTM, holo-tomographic microscopy; mESC, mouse embryonic stem cell; RI, refractive index. (TIFF) [file pbio.3000553.s008.tiff]

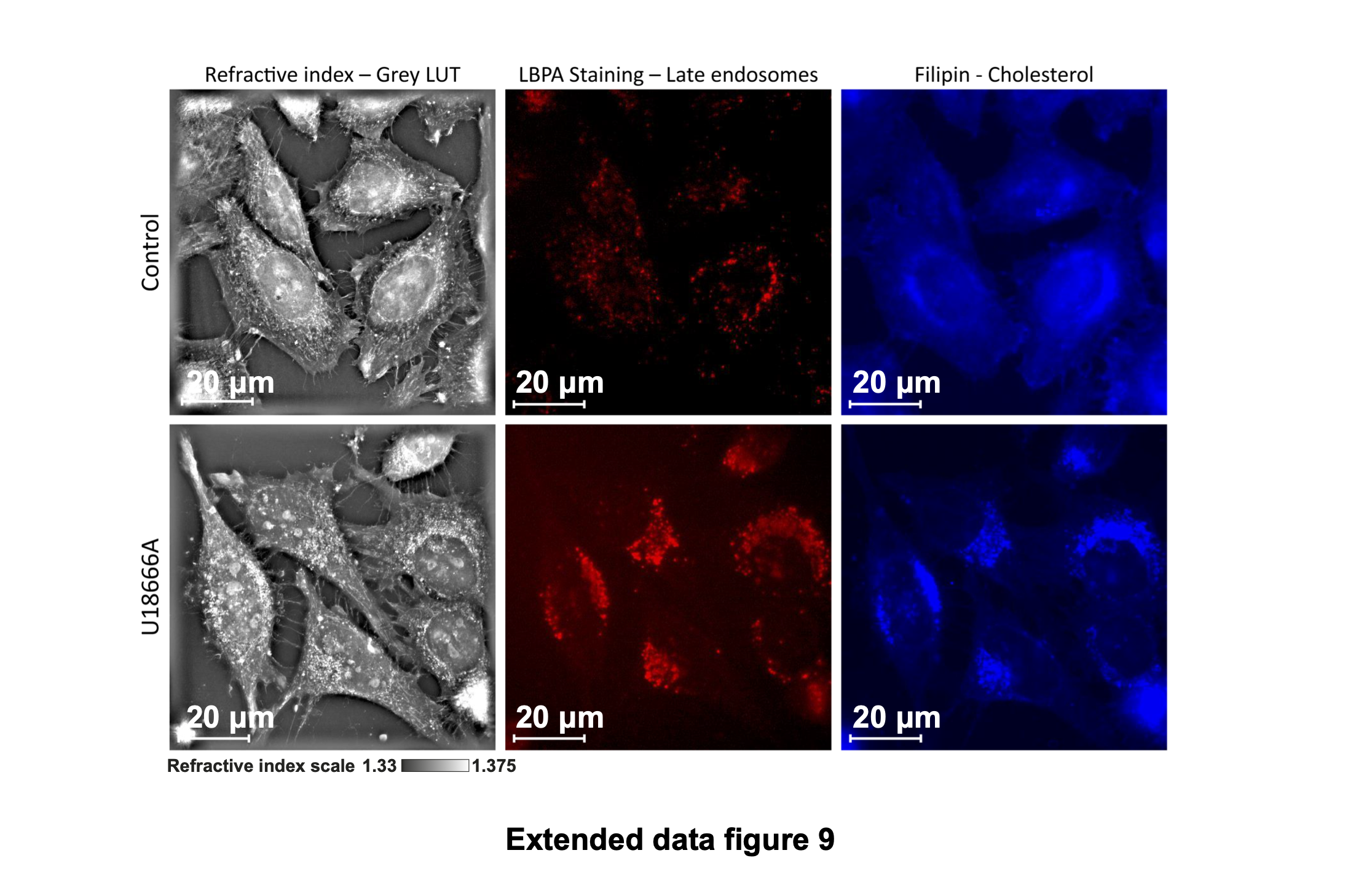

Supplement: S9 Fig — Compared to control, cells treated with U18666A show different perinuclear structures in the RI map that overlap with LBPA immunostaining and cholesterol filipin staining, indicating that the accumulation of cholesterol-rich late endosomes after treatment is observable in the RI map. LBPA, XXX; RI, refractive index. (TIFF) [file pbio.3000553.s009.tiff]

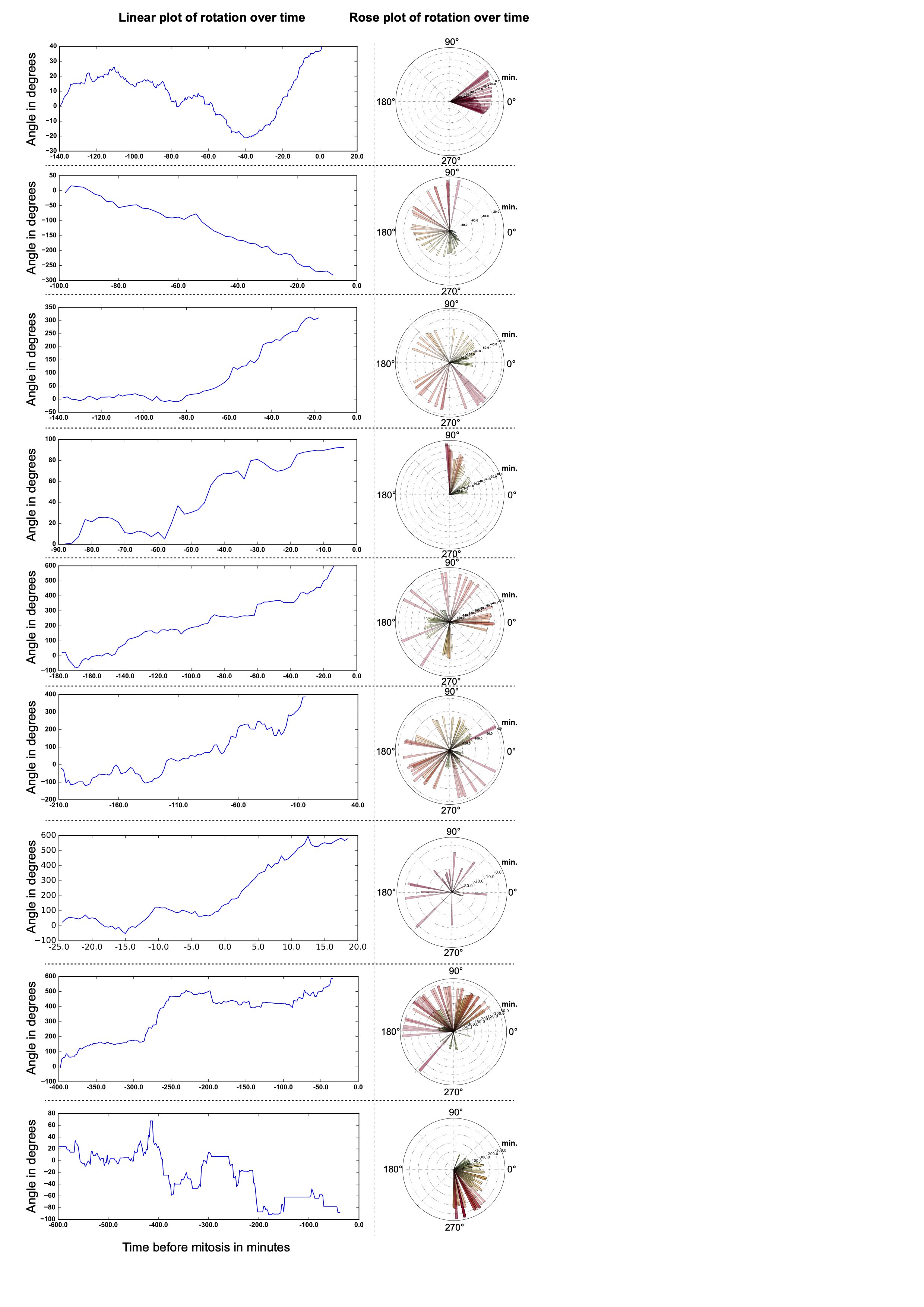

Supplement: S10 Fig — Plot and rose plot of intracellular rotation over time show the direction, extent, accelerations, and pauses of the observed rotation (related to S5 Movie). (TIFF) [file pbio.3000553.s010.tiff]
