# Supplementary material for: Image-based analysis of living mammalian cells using label-free 3D refractive index maps reveals new organelle dynamics and dry mass flux
Source: PLoS Biol. 2019 Dec 19;17(12):e3000553. doi: 10.1371/journal.pbio.3000553 (PMC6922317; doi:10.1371/journal.pbio.3000553)
Supplement: Attachment — Submitted filename: PBIOLOGY-D-19-02724 - edits from Academic Editor.pdf [file pbio.3000553.s019.pdf]

PLOS Biology

Image-based analysis of living mammalian cells using label free 3D refractive index maps reveals new organelle dynamics and dry mass flux

--Manuscript Draft--

**Manuscript Number:**	PBIOLOGY-D-19-02724
**Full Title:**	Image-based analysis of living mammalian cells using label free 3D refractive index maps reveals new organelle dynamics and dry mass flux
**Article Type:**	Initial Research Submission
**Corresponding Author:**	Mathieu Frechin, Ph.D.<br>Nanolive SA.<br>Eccublens, SWITZERLAND
**First Author:**	Patrick A. Sandoz
**Order of Authors:**	Patrick A. Sandoz
	Christopher Tremblay
	F. Gisou van der Goot
	Mathieu Frechin, Ph.D.
**Abstract:**	Holo-tomographic microscopy (HTM) is a label-free microscopy method reporting the fine changes of a cell's refractive indexes (RI) in 3D at high spatial and temporal resolution. By combining HTM with epifluorescence, we demonstrate that mammalian cellular organelles such as Lipid droplets and mitochondria show specific RI 3D patterns. To go further, we developed a computer vision strategy using FIJI, CellProfiler3 and custom code that allows to use the fine images obtained by HTM in quantitative approaches. We could observe shape and dry mass dynamics of lipid droplets, of endocytic structures, or of entire cells division that were so far and to the best of our knowledge out of reach. We finally took advantage of the capacity of HTM to capture the motion of many organelles at the same time to report a multi-organelle spinning phenomenon and study its dynamic properties using pattern-matching and homography analysis. This work demonstrates that HTM gives access to an uncharted field of biological dynamics and describes a unique set of simple computer vision strategies that can be broadly used to quantify HTM images.
**Additional Information:**	
**Question**	**Response**

Powered by Editorial Manager® and ProduXion Manager® from Aries Systems Corporation

15th of September 2019

Dear PLOS Biology editorial board,

We hereby submit to PLOS Biology the manuscript by Sandoz & Tremblay et al. entitled "**Image-based analysis of living mammalian cells using label free 3D refractive index maps reveals new organelle dynamics and dry mass flux**". As requested by Dr. Alvarez-Garcia, this work is a refoundation of **our previous Methods and Resource work (pbio.2006566)**. I sincerely thank the PLOS Biology editorial board for allowing us to strengthen our study. We demonstrate here the ground-breaking nature of holotomography for quantitative cell biology research. This microscopy technique works in 3D, at high-resolution and is non-invasive. We use it to observe unique mammalian dynamics at the cellular and subcellular levels. Since holotomography reports refractive index distributions in cells, the resulting images contains multiple biological structures simultaneously. In order to work with such complex but rich images we developed a unique set of computer vision procedures. By doing so we are bringing holotomography into the world of quantitative cell biology.

The computational aspect of the present study was absent from the previous version of the work. We applied it both on the original set of data and on new experimental data. Both computational and experimental efforts were done to go beyond the editorial and reviewer's requests, a document with point by point answers to those requests accompanies the submission.

Live cell imaging is a major challenge in biology: current high-content microscopy techniques damage the observed cells, that are at best fundamentally perturbed by the observation procedure. But the holotomographic device in this study works with extremely low power coherent light (hundred times lower than light sheet microscopy, ~0.01 nW/μm^2 versus ~1 nW/μm^2), providing an unmatched capability to perform live cell imaging at high temporal resolution, without phototoxicity, while observing a multitude of biological structures simultaneously.

We demonstrate here by comparing refractive index maps with fluorescent signals, that specific cellular organelles can be imaged alive in 3D, label-free and at high resolution using holotomography. Such capabilities allow us to shine light on unique lipid droplets dynamics, trafficking perturbations or organellar rotations. By doing so we bring new experimental, microscopic and computational resources and methods to perform live cell imaging at high temporal resolution and discover new dynamics on a wide range of biological objects.

I would like to provide some context to explain the delay since our last submission. We developed computational approaches and performed experiments to strengthen every aspects of our study; those tasks required significant time. Besides, I became a dad. This event had a slight impact on our schedules! This delay shows how much we value the opportunity you provided to us and is a testimony of our determination in providing the highest quality of work.

It is finally important to mention that the first version of this work that we proposed to you last year has been shown to the public as a preprint on BioRxiv (https://biorxiv.altmetric.com/details/47638569) with great success as we are in the top 5% of the most popular publications monitored by Altmetrics.

For its biological and technological and computational content, we believe that this work will be of great interest to the broad readership of PLOS Biology.

For conflict of interest reasons, we would like to exclude the Pr. YongKeun Park from the Biomedical Optics Laboratory at KAIST.

Suggested reviewers are:

-Pr. Tobias Walther, twalther@hsph.harvard.edu, expert of lipid droplets and metabolism.

-Pr. Petra Schwille, schwille@biochem.mpg.de, expert of membrane compartments.

-Pr. Guenter Albrecht-Buehler, g-buehler@northwestern.edu, expert in cell biology, observed the first nuclear rotation in 1988.

We look forward to hearing from you

Best regards,

Mathieu Fréchin

On behalf of all the authors.

Editorial requests:

In particular, we will need you to provide a full and scholarly description of the working principle of the HTM technology, including a figure and a detailed description showing how the instrument is set up,

First of all, we would like to express our gratefulness for providing a constructive feedback and for allowing us to submit a new version of the manuscript. We provide in the introduction of the current manuscript a fully referenced description of the fundamental principles of HTM. Furthermore, we disclose all the details of the HTM setup used in the study, from the laser source to the camera we use, in particular we explain how micromirrors are key to the quality of our HTM images and provide all the necessary literature. We omitted no details of the microscopic device, both at the theoretical and technological levels.

and an explanation of how the absolute value of refractive index and index of refraction maps were obtained.

The manuscript provides state-of-the-art details and necessary references to understand how refractive index and index of refraction maps are obtained. Please note that fully developing the mathematics and algorithms behind such process is not possible in the format of the manuscript and has been extensively developed in previous reviews and publications, we provide all the necessary references for those interested in such information.

We would also need you to respond to all the other points raised by the reviewers with, wherever appropriate, changes to the text and the figures.

We provide a full point by point answer to reviewers' comments in the submission files.

We would like you to provide a thorough study of lipid droplet biogenesis or the rotating nuclei that would justify the title of the article.

We believe that the current manuscript addresses this request for both the lipid droplet and organellar rotation topics. We greatly extended the quantification of lipid droplet growth and calculated dry mass fluxes within lipid droplets thanks to the fine segmentation of each lipid droplets and measurement of refractive index over time. This shines light on previously unknown dynamics and support the model of a fluid connectivity between existing and nascent lipid droplets through the ER membrane.

The phenomenon of organellar rotations that we report in this work is poorly known and likely very complex, in order to pave the road toward a better understanding of it, we expanded our data set and data analysis and developed a computer-vision approach in python to help quantify the characteristics of complex patterns motions in an unsupervised way, whether it is rotation in this case or any type of motion. This method is based on the use of scale-invariant features transform (SIFT) descriptors originally developed for matching different views of an object or of a scene. We then use homography matrices to calculate the rotation angles over time. Our quantifications suggest that the phenomenon can vary greatly in time, from 10 hours to 45 minutes, and in rotation magnitude, from 80 to 700 degrees, demonstrating that our approach can catch subtle nuclear movements as slow at 0.2 degree.min^{-1} or relatively quick rotations as fast as 13 degrees.min^{-1}. Nuclear rotations have recently been shown to be involved in the dormant state of mouse oocytes or to be key in migration of fibroblast, in this context, we expect our approach and observations to be essential.

Finally, we will need you to provide full disclosure of all real or potentially perceived competing interests that you might have.

I have fully disclosed it in the related field during the submission process. I am an employee and a minor shareholder of Nanolive SA who produces the microscope used in the study.

Point by point answers to reviewers' comments

Rev.1

1:

In this manuscript, the authors described an interesting study to use holo-tomographic microscopy (HTM) to map refractive index distribution of living cells free of labeling. Based on the intrinsic index of refraction contrast, lipid droplets inside the cells can be identified and correlated well with the fluorescence microscope. Live imaging of mouse embryonic stem cells was performed and an interesting pre-mitotic clockwise rotation of the cell organelles was observed. While the figures and videos provided are interesting, descriptions in the manuscript were not very clear. It is also not clear what new biological insights one can obtain from these label-free measurements.

We would like first to thank reviewer one for his constructive comments, they were very helpful in conducting the revision of our work. We believe the present manuscript provides extensive description of the rotation phenomenon thanks to new videos, and a full new computer vision strategy to analyze complex patterns motions. We also introduced thoroughly the phenomenon in its bibliographical context, extended our biological investigations, and discussed the impact and importance of our work in regard of the state of the art. Please consider that such process is poorly known, and only few rotations have been observed in the literature so far, and in very qualitative ways. Even if our 10 fully quantified rotations might feel a small number, and it is in fact a small number for drawing conclusions such as is it preferentially right or left handed rotations, it is still a big jump forward. Please consider also the technical challenge of recording such unique movies and the effort for creating a computational strategy to analyze them.

In addition to a full study of the organellar rotation, we also extended our study of lipid droplets, endosomal trafficking perturbation and introduced an analysis of stem cell division.

Specifically:

1. The working principle of the HTM technology is not clearly explained. Please include detailed description of the method and supplementary materials section to clearly explain how the instrument is set up and how the index of refraction maps are obtained. In particular, how is it different from phase contrast microscope and other optical coherence tomography or microscopy? How was the depth information obtained?

We provide in the introduction of the current manuscript a fully referenced description of the fundamental principles of HTM. Furthermore, we disclose all the details of the HTM setup used in the study, from the laser source to the camera we use, in particular we explain how micromirrors are key to the quality of our HTM images and provide all the necessary literature. We omitted no details of the microscopic device, both at the theoretical and technological levels. The manuscript provides state-of-the-art details and necessary references to understand how refractive index and index of refraction maps are obtained, this includes a scholarly description of how the depth can be acquired thanks to the tomographic aspect of the microscope. Please note that fully developing the mathematics and algorithms behind such process is not possible in the format of the manuscript and has been extensively developed in previous reviews and publications, we provide all the necessary references for those

interested in such information, in particular for understanding the fundamental differences between the various existing label-free microscopy techniques. Please understand that such comparisons are the matter of complete reviews, that are referenced in the manuscript. There is however a quick description of other label-free techniques in parallel with holotomography. This should however just provide a rough idea of the differences and would not succeed in providing all the fundamentals. Finally, the topic of the manuscript are not the concepts of the microscopy technique itself that have been developed previously in dedicated publications, but its applications and the revolution it represents for live cell imaging and quantitative cell biology.

2. Was the absolute value of refractive index (RI) map obtained from HTM or a relative RI map? Fig. 2 shows signal profile comparisons between HTM and fluorescence microscopy. But all the signal was normalized. How was the normalization done? Can absolute RI be obtained from the measurement?

We do measure absolute values of refractive index. The manuscript contains a direct explanation of it and link to relevant bibliography for going more in depth with the theoretical concept allowing to retrieve refractive index maps. The normalization of the profile is done the simplest way, the minimal value of the shown profiles is set to 0 and the maximum to 1, and this for RI and Fluo signals.

3. It can be seen that the nuclei show clear clusters in the HTM images. How is the RI contrast of nuclei compared to lipid droplets? One related question is that how can be HTM measurements be more specific? Those clear clusters are nucleoli, they have a smaller RI than lipid droplets, we are extremely sorry but we do not understand what reviewer 1 means by "how can be HTM measurements be more specific", the contrast is provided by the RI of the structure and not by a chemical or genetic fluorescent dye that could in fact be partially unspecific. We believe that the new version of the manuscript might contain the information reviewer 1 was looking for as it contains much more material.

4. Please mark important features in the figures and clearly explain what we are looking at. We are really sorry but this point was unclear to us, we took that comment into account and applied it in our making of the new figures the way we could interpret it, we believe that this has been addressed in the present manuscript

5. The descriptions of the supplementary figures and videos are very short and unclear. Please provide detailed descriptions in the supplementary document.

We believe that this has been entirely reworked and addressed in the present manuscript

6. In Fig. 2, how was the 3D profile obtained?

The refractive indexes of mitochondria and lipid droplets, as well as the way they organize in space, are clear signatures of the organelles that can be isolated based on their RI values and simply displayed as a 3D structure in FIJI.

7. In Fig. 3, no images for the control experiments were shown. Please add them. Also, how were the error bars obtained from just a single experiment?

This figure has been removed and the analysis completely made anew with explanations in figure legends and material and methods.

8. In Fig. 4, why are all the organelles move clock-wise? It is not clear.

We believe the present manuscript provides extensive description of the rotation phenomenon thanks to new videos, and a full new computer vision strategy to analyze complex patterns motions. We also introduced thoroughly the phenomenon in its bibliographical context, extended our biological investigations, and discussed the impact and importance of our work in regard of the state of the art. Please consider that such process is poorly known, and only few rotations have been observed in the literature so far, and in very qualitative ways. Even if our 10 fully quantified rotations might feel a small number, and it is in fact a small number for drawing conclusions such as is it preferentially right or left handed rotations, it is still a big jump forward. Answering the question of why it moves clock-wise is a matter of long cellular and molecular investigations, and we are certain that our contribution is a first essential step towards such understanding.

9. What new biology do we learn from these live cell imaging experiments? Why are they important?

This broad comment is addressed by the whole manuscript.

10. Please discuss what the limitations of HTM are. Not all cellular organelle can be clearly differentiated. The specificity may be low. Also, 3D image capability may also be limited.

The limitations and capabilities of HTM is the topic of the first chapter of the results in the manuscript, we demonstrate that 3D is of good quality, and identify clearly strengths and limitations of HTM. Here is an exert of it: “We next used the HTM/epifluorescence setup to compare the RI sections and the corresponding fluorescent signals of the cells under investigation. We first analyzed the largest organelle in the cell, namely the endoplasmic reticulum (ER) using KDEL-GFP as a fluorescent marker. Analysis of the corresponding RI map (S4A Fig), and the quantitative evaluation of RI and fluorescent signals correlations (S5A and B Fig) indicated that the ER does not lead to a RI signal in control cells. Similarly, using NAGTI-GFP as a fluorescent marker, we found that the Golgi apparatus is not detected by HTM (S4B Fig). The fact that the ER and the Golgi are “silent” in terms of RI mapping is advantageous given that the ER is present throughout the cell and its detection would have hampered the proper analysis of any other organelle. In contrast, labeling of cells with the mitochondrial marker Mito-YFP (Fig 2A) or lipid marker Bodipy (Fig 2B) revealed that mitochondria and lipid droplets provide a clearly distinguishable RI signal in 2D and 3D.”

We are again sorry to not understand exactly what reviewer 1 means by “specificity may be low” in the context of HTM as this technique works label-free, and therefore is not subjected to specificity problem due to staining unspecific signals and reports an inherent quality of cellular and subcellular objects, their refractive index.

Rev. 2:

This manuscript reads like an advertisement for the 3D Cell Explorer Fluo, which is a commercial instrument developed by Nanolive, LLC. Nanolive is a start-up company spun out of EPFL and employs most of the authors, as is acknowledged in the financial disclosure.

We thank reviewer 2 for the time invested in writing the comments, it motivated us and contributed to make the present manuscript and we are grateful for that. However, we can only strongly disagree with the point of view of referee 2 as it is a subjective comment about the motivations of Nanolive's scientists. Not acknowledging that the work was made in collaboration with the laboratory of Pr. Gisou Van der Goot at the EPFL, a world-wide leading laboratory in the field of membrane and organelle biology that has no interest in Nanolive, is not fair.

The microscope's operating principle is described in Ref. [6], but is not explained in the present manuscript.

We provide in the introduction of the current manuscript a fully referenced description of the fundamental principles of HTM. Furthermore, we disclose all the details of the HTM setup used in the study, from the laser source to the camera we use, in particular we explain how micromirrors are key to the quality of our HTM images and provide all the necessary literature. We omitted no details of the microscopic device, both at the theoretical and technological levels. The manuscript provides state-of-the-art details and necessary references to understand how refractive index and index of refraction maps are obtained.

This causes concern because the introduction asserts that the Nanolive instrument improves upon other implementations of the technology by having a "completely scattering-free optical setup." The manuscript does not explain how this is achieved,

We rewrote this part of the manuscript, and provided the necessary references to explain how HTM systems can suffer from coherent noise created by scattered light from the rotational scanning mechanism, it is important to note that our manuscript aim at explaining how holotomography can help cell biologists, and not to compare holotomographic devices. We believe it is important that each researcher makes his own opinion on the quality of the images he can obtain from various devices. We provide only an educated view on the important factors impacting image quality in holotomography.

nor does it explain why the deficiencies in other implementations would have prevented the observations reported here.

I reiterate here that our goal is to show the gain HTM can provide to cell biologists, as a matter of fact, all HTM devices should benefit from our work.

The principal measurements reported in this manuscript are presented as visualizations of the spatial variation of the refractive index. Although the precision for refractive index measurement is reported to be "2.71E-04" (note the incorrect formatting) the actual scale of the refractive index is not reported for any of the images. What is the refractive index of the lipid droplets, for example? I would assume it is around 1.45 at the imaging wavelength.

We apologize for not understanding properly this remark of reviewer 2, while lipid droplets do have a RI around 1.41-1.42, what actually matters in the context of our study is that we could accurately detect them over space and time, and it is the case, as proven in the manuscript. We would like to reiterate that our goal here is to put HTM in a true quantitative biology context, to avoid confusion at this level we rewrote the

related part and removed the reference about precision since we believe that our biological images translate perfectly what the microscope can or cannot do.

Results for the spatial resolution reported in Fig. 2 recapitulate resolution measurements from Ref. [6].

We are sorry but do not understand that comment, as there is nothing similar to be found in term of biological material, images, or quantifications.

The remainder of the manuscript presents a collection of nice imaging results in several different systems that highlight the ability of the technique to track intracellular structures over long periods without chemical staining. Each subject is treated in one or two paragraphs that highlight the capabilities of the technique without substantially advancing our understanding of the systems being studied. This culminates with the discussion of the data in Figure 4 describing circulation within cells before mitosis. None of these topics is discussed in depth, and indeed the authors conclude the narrative by explaining that they serve to demonstrate the use of HTM for biological applications.

We would like to thank reviewer 2 for this criticism, again, while we do not agree with the idea that it is simply advertisement material, this broad comment triggered a profound effort to make the study better, we believe that this broad point is now addressed by the whole new manuscript.

The title and concluding sentences propose that premitotic circulation is the focus of the paper. This simply is not the case. The phenomenon is observed, but not studied systematically, and little about it is learned. A thorough study of this, or any of the other examples could well be valuable contributions. This present manuscript serves mostly to highlight what the instrument can do, which is not sufficient justification for publication.

The phenomenon of organellar rotations that we report in this work is poorly known and likely very complex, in order to pave the road toward a better understanding of it, we expanded our data set and data analysis and developed a computer-vision approach in python to help quantify the characteristics of complex patterns motions in an unsupervised way, whether it is rotation in this case or any type of motion. This method is based on the use of scale-invariant features transform (SIFT) descriptors originally developed for matching different views of an object or of a scene. We then use homography matrices to calculate the rotation angles over time. Our quantifications suggest that the phenomenon can vary greatly in time, from 10 hours to 45 minutes, and in rotation magnitude, from 80 to 700 degrees, demonstrating that our approach can catch subtle nuclear movements as slow at 0.2 degree.min^{-1} or relatively quick rotations as fast as 13 degrees.min^{-1}. Nuclear rotations have recently been shown to be involved in the dormant state of mouse oocytes or to be key in migration of fibroblast, in this context, we expect our approach and observations to be essential.

Manuscript Click here to download Manuscript Sandoz-et-al-HD-plos2019-1-withfigures.pdf

Image-based analysis of living mammalian cells using label free 3D refractive index maps reveals new organelle dynamics and dry mass flux

Patrick A. Sandoz[2]†, Christopher Tremblay[1,2]†, F. Gisou van der Goot[2*], Mathieu Frechin[1*]

[1] Nanolive SA, Ecole Polytechnique Fédérale de Lausanne (EPFL) Innovation Park, Chemin de la Dent d'Oche 1A, 1024 Ecublens, Switzerland

[2] Global Health Institute, Life Sciences Faculty, EPFL, Station 19, CH-1015 Lausanne, Switzerland

†: contributed equally to this work

*corresponding author / Contact: gisou.vandergoot@epfl.ch / mathieu@nanolive.ch

Abstract

Holo-tomographic microscopy (HTM) is a label-free microscopy method reporting the fine changes of a cell's refractive indexes (RI) in 3D at high spatial and temporal resolution. By combining HTM with epifluorescence, we demonstrate that mammalian cellular organelles such as Lipid droplets and mitochondria show specific RI 3D patterns. To go further, we developed a computer vision strategy using FIJI, CellProfiler3 and custom code that allows to use the fine images obtained by HTM in quantitative approaches. We could observe shape and dry mass dynamics of lipid droplets, of endocytic structures, or of entire cells division that were so far and to the best of our knowledge out of reach. We finally took advantage of the capacity of HTM to capture the motion of many organelles at the same time to report a multi-organelle spinning phenomenon and study its dynamic properties using pattern-matching and homography analysis. This work demonstrates that HTM gives access to an uncharted field of biological dynamics and describes a unique set of simple computer vision strategies that can be broadly used to quantify HTM images.

Introduction

Because of the transparent nature of a cell, microscopy techniques either use fluorescent markers or transform optical properties of the sample into an observable contrast (*e.g.* phase contrast, DIC). Each of these techniques comes with limitations. Photobleaching, phototoxicity, interference of markers or ectopically expressed engineered proteins are major concerns. Classical label-free imaging techniques, while less perturbing, provide images with low information content due to poor contrast and resolution. In this context, holo-tomographic microscopy (HTM)(1) is of great interest, as it can provide label-free, high-content images using very low power light source that generates no phototoxicity. The HTM device used in this study is based on quantitative phase microscopy (2–5) where the object's complex wave field is encoded into a hologram. A partially coherent light beam generated by a laser diode (520 nm) is split in two beams to create a Mach-Zehnder interferometer setup(6); the first one, called the object beam, interacts with the sample before being collected by a 60x objective, while the second one is the reference beam. The two beams are later brought to interference and the resulting hologram is recorded on a CMOS camera(1–4). Moreover, the device combines this classical holographic approach with rotational scanning of the specimen(5) using a rotating arm equipped with a mirror as described in Fig 1A. The synthesis of the rotational series of scattering spectra is achieved using complex field deconvolution(7–9) in order to reconstruct a full 3D refractive index (RI) tomogram of even live(10) sample's. Importantly, dynamically adjustable mirrors allow the optics of the HTM device to self-adjust during an acquisition experiment in order to adapt to sample changes such as medium evaporation(11). While RI distributions start to be used in life sciences studies(12–15) and specific refractive index signatures for cell structures(16,17) have been partially analyzed, HTM systems can suffer from coherent noise created by scattered light from the rotational scanning mechanism(18). This coherent noise perturbs the quality of the generated holograms and impedes spatial resolution, which results in reduced RI sensitivity and perceived image quality(19). Thanks to its rotational scanning mechanism made of a rotating mirror, the HTM setup used in this study overcomes this limitation and allows, to the best of our knowledge, a unique characterization of cellular and organelle details by refractive index in space and time. The next challenge was to go beyond a qualitative approach and to use computer assisted image analysis in order to harness the full potential of HTM images. We developed computer vision strategies using FIJI(20), CellProfiler3(21) and custom code in order to investigate the evolution of number, shape and dry mass flow of lipid droplets and endocytic structures as well as of full mouse embryonic stem cells over division. Thanks to the inherent multiplexing capacity of HTM, *i.e.* its capacity to capture multiple organelles and cellular structures all at once, we could finally observe a multi-organelle rotation within mammalian cells. In order to characterize this phenomenon and pave the road for more in-depth studies of its molecular origins and role for the cell, we developed a computer vision strategy adapted to quantify the rotation of complex patterns using pattern-matching and homography(22,23).

Results & Discussion

Identification of cellular organelles at high resolution using holotomographic microscopy

Our HTM device is equipped with simultaneous holotomography and epifluorescence imaging (Fig 1A), we could therefore compare fluorescence imaging of subcellular markers with Refractive Index (RI) maps to determine whether specific organelles have a recognizable RI signature that would allow label-free analysis of organelle biology in control conditions or upon perturbations. Firstly, HTM performances were compared to other label-free methods, fixed HeLa cells were imaged with bright-field microscopy where contrast is created by the attenuation of the transmitted light, or phase contrast(24) where contrast is created by the capacity of the sample to scatter light, or differential interference contrast (DIC)(25) that relies on two rays of polarized light, a reference and a sampling ray, that interfere to create contrast, and finally HTM (S1 Fig). The plasma membrane and to a lesser extent lipid droplets (LDs) and nuclei could be distinguished by the established label-free technologies but were perfectly observable in 2D and 3D images provided by HTM (Fig 1B). LDs were also visible by HTM in U2OS, Fibroblasts, RPE1, MEF, Hepa1.6, HAP1 and 3T3 cells differentiated in adipocytes (S2 Fig). In addition to LDs, HTM could clearly discriminates (in 2D and 3D) other intra-cellular details that could not be distinguished at all by the other methods (Fig 1B, S1 Fig) such as what we identify later as mitochondria. It is important to note that dehydrating fixation methods affect organelles structural integrity, altering their RI and thus the image quality when observed with HTM. Paraformaldehyde however is a viable solution for observing fixed cells with HTM as it allowed the structural preservation of subcellular details (S3 Fig).

rotational

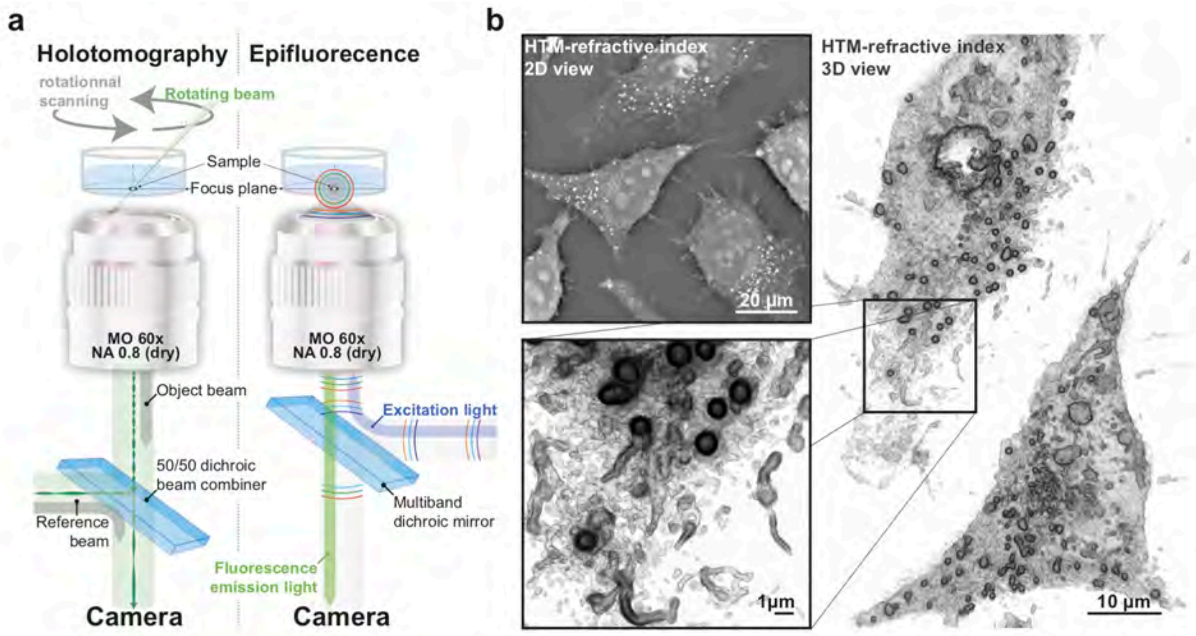

Figure 1 | Holotomographic microscopy of subcellular structures. a, Scheme of the holotomographic microscopy (HTM) setup coupled to epifluorescence used in this study. **b,** 2D and 3D images of unperturbed HeLa cells taken with HTM.

We next used the HTM/epifluorescence setup to compare the RI sections and the corresponding fluorescent signals of the cells under investigation. We first analyzed the largest organelle in the cell, namely the endoplasmic reticulum (ER) using KDEL-GFP as a fluorescent marker. Analysis of the corresponding RI map (S4A Fig), and the quantitative evaluation of RI and fluorescent signals correlations (S5A and B Fig) indicated that the ER does not lead to a RI signal in control cells. Similarly, using NAGTI-GFP as a fluorescent marker, we found that the Golgi apparatus is not detected by HTM (S4B Fig). The fact that the ER and the Golgi are "silent" in terms of RI mapping is advantageous given that the ER is present throughout the cell and its detection would have hampered the proper analysis of any other organelle. In contrast, labeling of cells with the mitochondrial marker Mito-YFP (Fig 2A) or lipid marker Bodipy (Fig 2B) revealed that mitochondria and lipid droplets provide a clearly distinguishable RI signal in 2D and 3D.

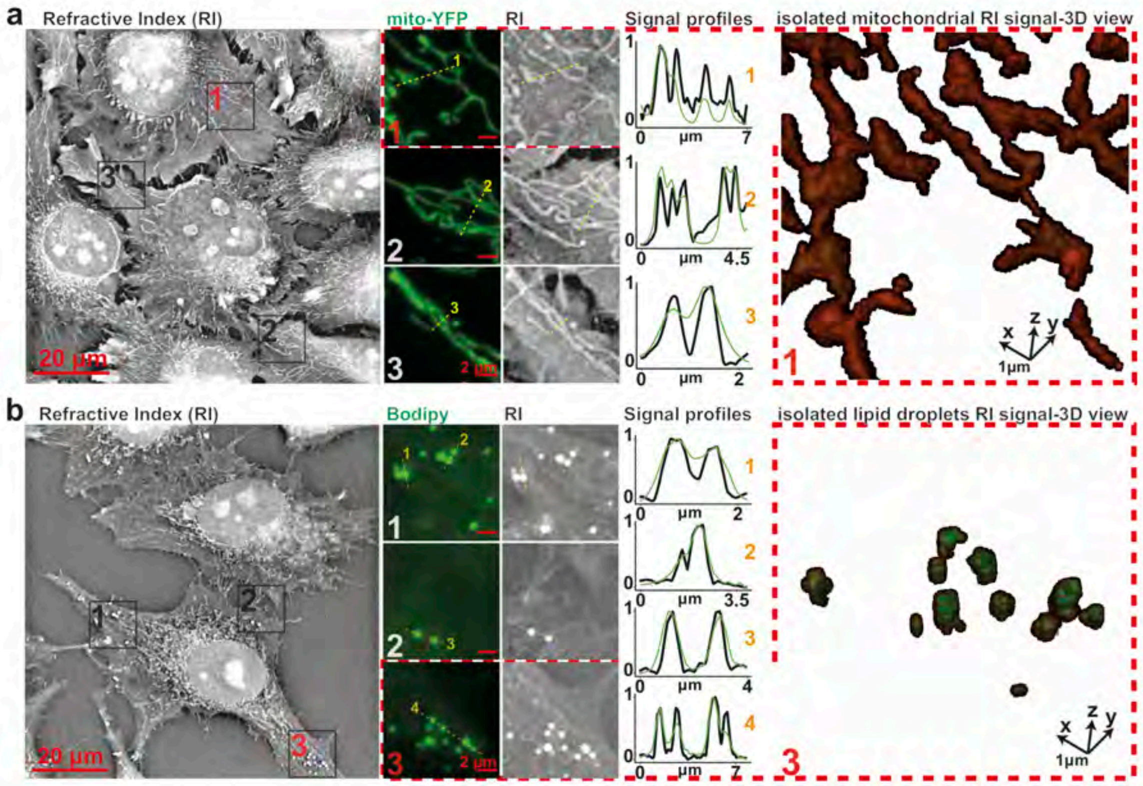

Figure 2 | Comparison of specific fluorescent signals and refractive index map. Visual and profile comparison as well as 3D view of **a,** HeLa cells refractive index (RI) map to mitochondria-specific fluorescent signal (mito-YFP) or **b,** to a lipid droplet-specific fluorescent signal (Bodipy) shows specificity and gain in resolving power. Profiles were normalized between 0 and 1 for proper scaling.

This visual conclusion was confirmed using Kolmogorov-Smirnov (S5A Fig) and Pearson coefficient tests (S5B Fig). It was also confirmed by the comparison of Mito-YFP and Bodipy fluorescent signals with independent human expert labelling of mitochondria and lipid droplets in RI maps that shows over 90% of overlap (S5C Fig), altogether validating a specific correlation of the RI and fluorescent signals.

Interestingly, while in epifluorescence (or in a microscope using the same optics), such subcellular structures are being diffraction limited to maximum of resolution of $\eta_{fluo}^{Abbe} \approx 325nm$, our HTM's theoretical resolution limit(10) allows for $\eta_{HTM}^{Abbe} \approx 162nm$. Accordingly, the signal profiles in Fig 2A and 2B show an improvement in resolving subcellular structures using our RI measurement over the conventional epifluorescence signal. Thus, the resolution of mitochondrial tubules down to 186 nm by the used HTM setup (S6 Fig) demonstrates a sub-diffraction imaging regime with respect to η_{fluo}^{Abbe}. To further confirm the ability of HTM to image mitochondria, we promoted mitochondrial fission by overexpressing the mitochondrial fission protein 1 Fis1(26). While cells expressing a mitochondria-targeted dsRed protein (S7A Fig) exhibit regular elongated and thin mitochondria, cells transfected with Fis1 presented perinuclear collapsed and round shaped structures (S7B Fig). We finally performed time-lapse acquisitions of mouse embryonic stem cells at a frequency of 4 image.min^{-1} in order to confirm our capacity to observe individual mitochondrial fission and fusion events (S8 Fig and S1 movie) over time lapse experiments. All together these investigations demonstrate that HTM is suited for the study of cellular organelles with great spatial and temporal resolution.

Image-based analysis of lipid droplet dry-mass dynamics

So far dynamics of LDs have been visualized using fluorescence microscopy and Coherent Anti-Stokes Raman Scattering (CARS and the variant SRS)(27,28). On one hand, Fluorescence microscopy coupled to specific bodipy dyes(27) provides contrast and specificity but is inherently phototoxic, forcing to mitigate acquisition length and frequency in order to reduce the perturbation of LDs dynamics. On the other hand, CARS allows to work label-free; however, it is a complex and costly technology that has specific constraints regarding signal specificity and temporal resolution(28). The consequence of those limitations is a lack of finely resolved dynamics of the first moments of LDs appearance and growth. HTM can provide such data and could participate to a better understanding of the still elusive process of lipid droplets formation(29). HeLa cells were incubated with oleic acid (OA) to trigger the growth of lipid droplets(30) while imaged using HTM at a regime of 1 RI volume.min^{-1} (S2 movie). All volumes were then projected into 2D RI images using FIJI (Fig 3A), a requirement to be able to use CellProfiler3's (CP3) object detection modules. CP3 was used for a systematic and precise segmentation of LDs objects over time (Fig 3B and C). Segmented objects where then used to extract the mean refractive index of each detected lipid droplet (LD) and we considered LDs as spheres in order to calculate their dry mass content based on linear calibration model (31). After three hours of incubation with OA, lipid droplets increased app. 16-fold in number (Fig 3B and D, top left plot) and each single LD contained app. 6 times more dry mass on average (Fig 3B and D, bottom left plot). Thanks to the extremely low light power used in our HTM setup, we could perform high frequency acquisitions without perturbing organelles biology and could highlight new dynamic features of cellular lipid accumulation.

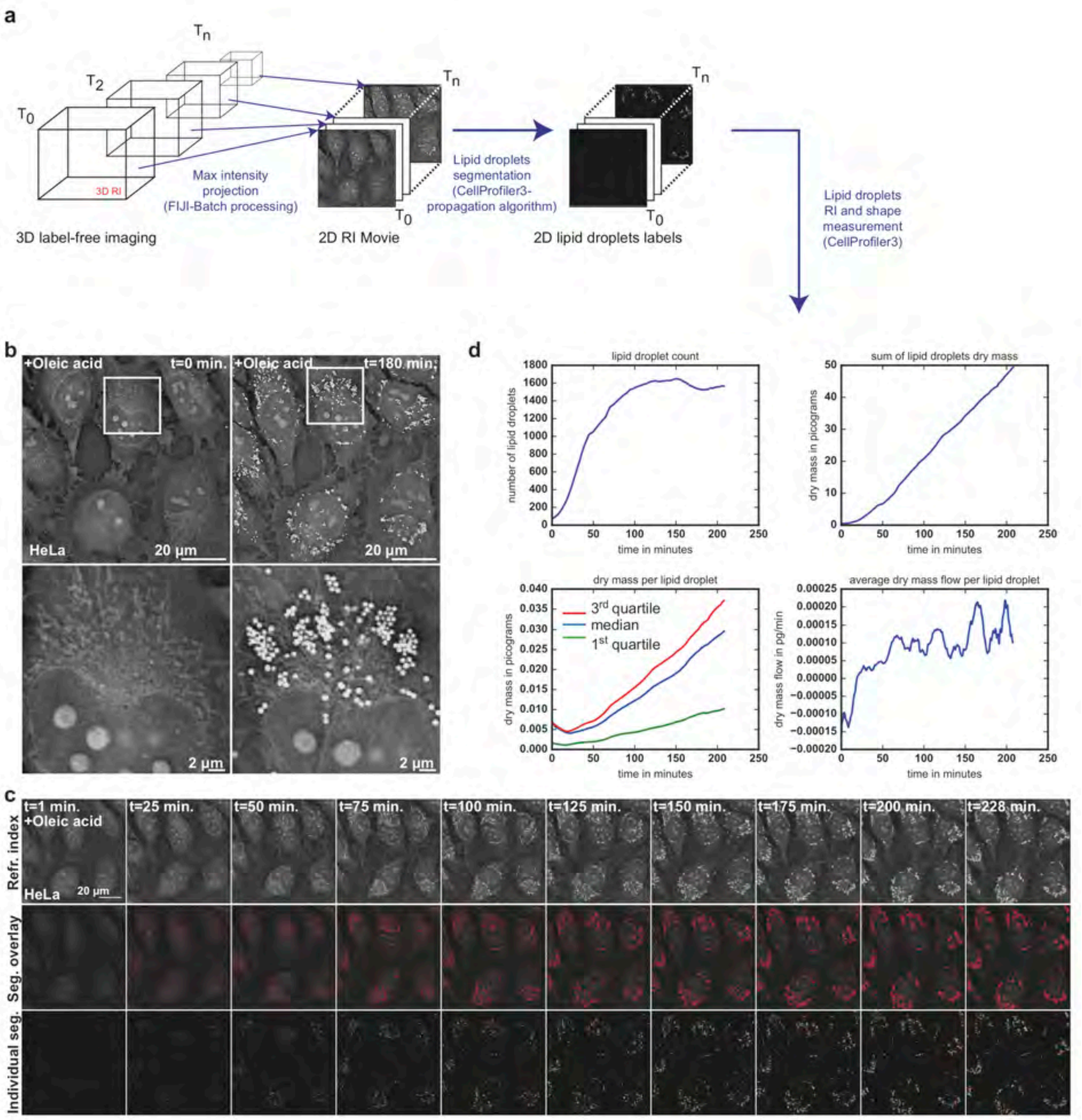

Figure 3 | Holotomographic live microscopy reveals lipid droplet dynamics. a, Refractive index (RI) map processing and analysis strategy **b,** Refractive index (RI) map of HeLa cells just before (t=0h) or three hours after (t=3h) loading with oleic acid (OA). **c**, Time series of HeLa cells 2D RI maps, overlay of lipid droplets (LD) segmentations and individual LD objects after treatment (OA) (related to S2 movie). **d,** Time traces of *i)* the increase of LD number in LD per minute, *ii)* the overall dry mass accumulation within LD or *iii)* per LD in picograms per minute and *iv)* average dry mass flow observed per LD in picograms per minute[2].

Firstly, the mean dry mass contained per lipid droplets decreases in the first 20 to 25 minutes after OA treatment with a mean dry mass flow per detectable lipid droplet starting around -1×10^{-4} $pg.min^{-1}.LD^{-1}$ and reaching a 0 net flow around 25 minutes to later stay positive around 1×10^{-4} $pg.min^{-1}.LD^{-1}$ (Fig3D, bottom plots). To give concreteness to such numbers and if we consider that only oleic acid had a net contribution to LDs growth in our experiment, 1×10^{-4} $pg.min^{-1}.LD^{-1}$ represents on average 2.1×10^{4} molecules of oleic acid processed and stored in each LD every minute.

Importantly, during these first 25 minutes we also see that the number of lipid droplets increases steadily (Fig3D, top left plot) and is multiplied app. 3-folds. We also observed that in our experiments new lipid droplets do not arise from the fission of pre-existing lipid droplets like it was reported in Yeast(32). We therefore see that OA loading on HeLa cells has a surprising effect, LDs become smaller but more numerous, suggesting strongly that preexisting LDs unload material into a common medium to prepare and feed new lipid storage units before growing them. This is to our knowledge a new observation that would support the model of a fluid connectivity between existing and nascent lipid droplets through the ER membrane(33–35).

'pool' is a better word choice than medium

We then observe that the number of lipid droplets reaches a plateau around 100 minutes, however the total dry mass content of all lipid droplets (LDs) increases until 210 minutes where OA loading starts to become toxic (36). This nearly linear accumulation hides bursts in the average dry mass flux per lipid droplet (Fig3D, bottom right plot, after 50 minutes). If lipid droplets would accumulate lipids asynchronously the resulting average flux curve would be smooth, since the random variations at single LD level at each time point would compensate each other's ; on the contrary, those bursts we observe in the average flux of dry mass per LD suggest a synchronized acceleration of lipid storage in at least a significant subset of lipid droplets. Such synchronization of fluxes could be achieved by sharing a common medium and are an additional argument in favor of a fluid connection of lipid droplets through the ER.

Image-based analysis of dry mass accumulation in the late endocytic pathway

The fact that mitochondria, which are dense in membranes, and lipid droplets are readily observable by HTM, led us to speculate that other lipid rich structures could provide a clear RI signal. We therefore induced the formation of a lipid rich organelle and did so by treating HeLa cells with U18666A, a charged amino derivative of cholesterol(37,38) that can trigger lipid and cholesterol accumulation in the late endocytic pathway and mimic the phenotype of the Nieman Pick Type C disease(38). We imaged HeLa cells for 10h after treatment with U18666A using HTM at a frequency of 1 image.min^{-1}, we observed the appearance of perinuclear organelles with a well-defined RI signal (Fig 4A, S3 movie). These structures are rich in cholesterol, as indicated by the filipin-positive staining, and co-localize with the late endosomal lipid LBPA (S9 Fig). The accumulation of these U18666A-induced structures was followed and quantified using the image analysis procedure developed for lipid droplets but adapted for smaller objects in CP3's detection modules (Fig 4B). The number of detectable lipid rich endocytic structures increased app. 6-folds over a 10 h treatment (Fig 4C, top left plot). This curve closely follows the total accumulation of dry mass in the structures (Fig 4C, top right plot), this is explained by the relative homogeneity in size of late endosomal structures over the whole experiment (Fig 4C, bottom left plot). Thanks to our high temporal resolution and computer-vision approach, we could observe details of dry mass fluxes inaccessible to the eye or to label-based time lapse approaches. This analysis reveals that the accumulation of lipid and cholesterol in late endosomal structures is the result of oscillating fluxes of material that successively accumulates and is removed. In accordance with current models of the effect of U18666A on lipid and cholesterol trafficking, our quantification suggests that U18666A do not strictly block recycling, since negative fluxes suggest a recycling of a part of the accumulated material. It rather suggests that U18666A perturbs the equilibrium between recycling and uptake as suggested before (38) leading to progressive accumulation in late endosomal structures. Our approach should prove extremely useful to study the contribution of given genes in the formation of aberrant late endosomal structures in lysosomal storage diseases or to find drugs able to fight lysosomal storage diseases such as Niemann–Pick type C disease.

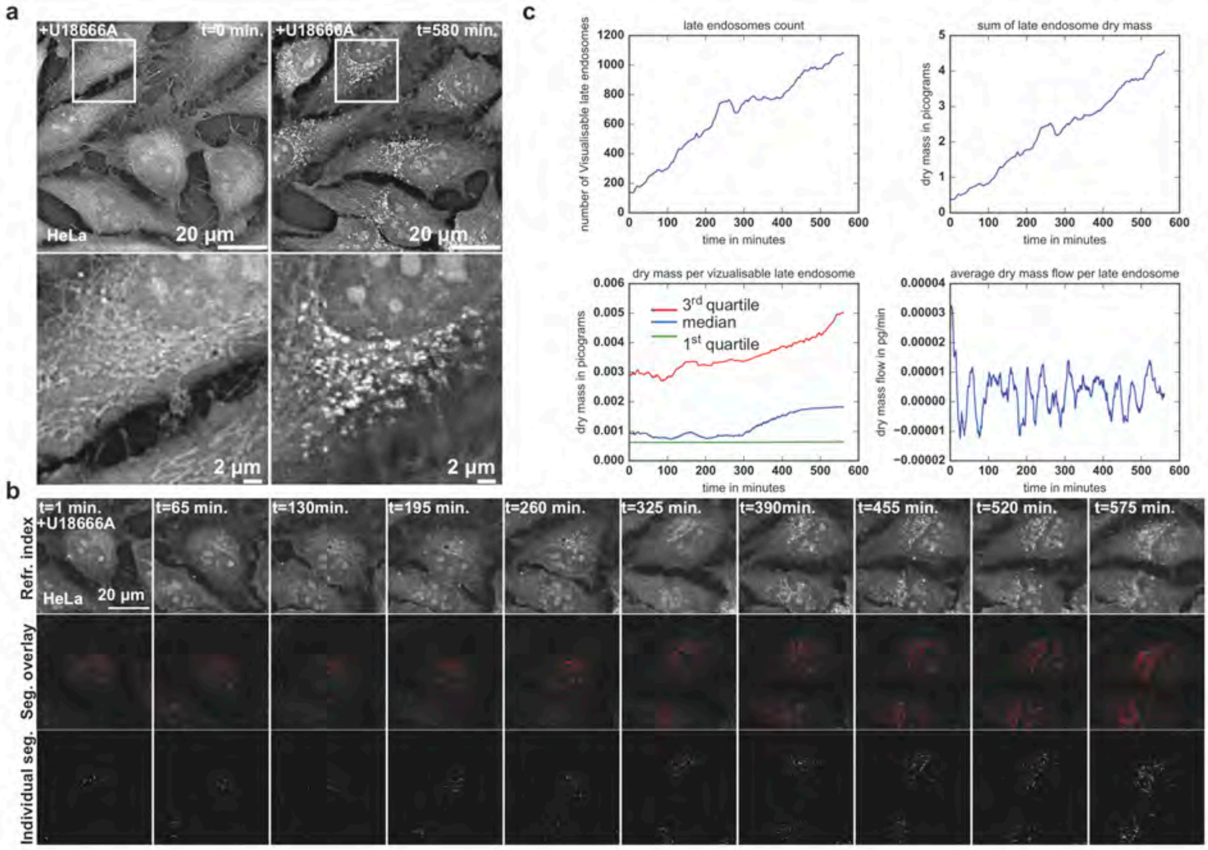

Figure 4 | Holotomographic live microscopy reveals perturbed trafficking dynamics. a, Refractive index (RI) map of HeLa cells just before (t=0h) or ten hours after (t=10h) treatment with the compound U18666A. **b**, Time series of HeLa cells 2D RI maps, overlay of late endosome (LE) segmentation and individual LE objects after treatment (U18666A) (related to S3 movie). **c,** Time traces of *i)* the increase of LE number in LE per minute, *ii)* the overall dry mass accumulation within LE or *iii)* per LE in picograms per minute and *iv)* average dry mass flow observed per LE in picograms per minute^2.

Image-based analysis of cellular dry mass dynamics before, during and after mitosis

We then investigated the potential of holotomographic microscopy combined with fine cell segmentation to study the volume and dry mass of mouse embryonic stem cells (mESCs) over mitosis. Such investigation is a technical challenge(39,40). Recent studies have investigated mammalian cell's volume over mitosis using a wide range of techniques from confocal microscopy to fluorescence exclusion method (41–44) but not with holotomography, which has the advantage of showing fine cellular details within high-quality refractive indexes (RI) maps directly in 3D. Some studies (43,44) show that mammalian cell's volume diminishes during mitosis while others(41) demonstrate that various mammalian cell lines can swell during mitosis. Even if they lead to contradicting results, those studies show the importance of a proper object detection and our approach focuses on improvements at that level thanks to HTM combined with state-of-the art object detection rather than concluding on mitotic volume dynamics, which would require a study on its own. We imaged mESCs at a frequency of 2 RI volume.min^{-1}, all volumes were projected into 2D RI images using FIJI (Fig 5A) in order to be able to use CellProfiler3's (CP3) much like the procedure developed for lipid droplets. Cell objects were detected using the propagation algorithm of CellProfiler3. This 2D object segmentation on projected RI maps was then reapplied on each 3D volumes, slice by slice, and adapted using the RI threshold defined during the 2D segmentation step (Fig 5B). This seeding procedure using a 2D pre-mask ensures a continuity over the z dimension since CellProfiler3 is not yet ready for 3D object detection over large data sets.

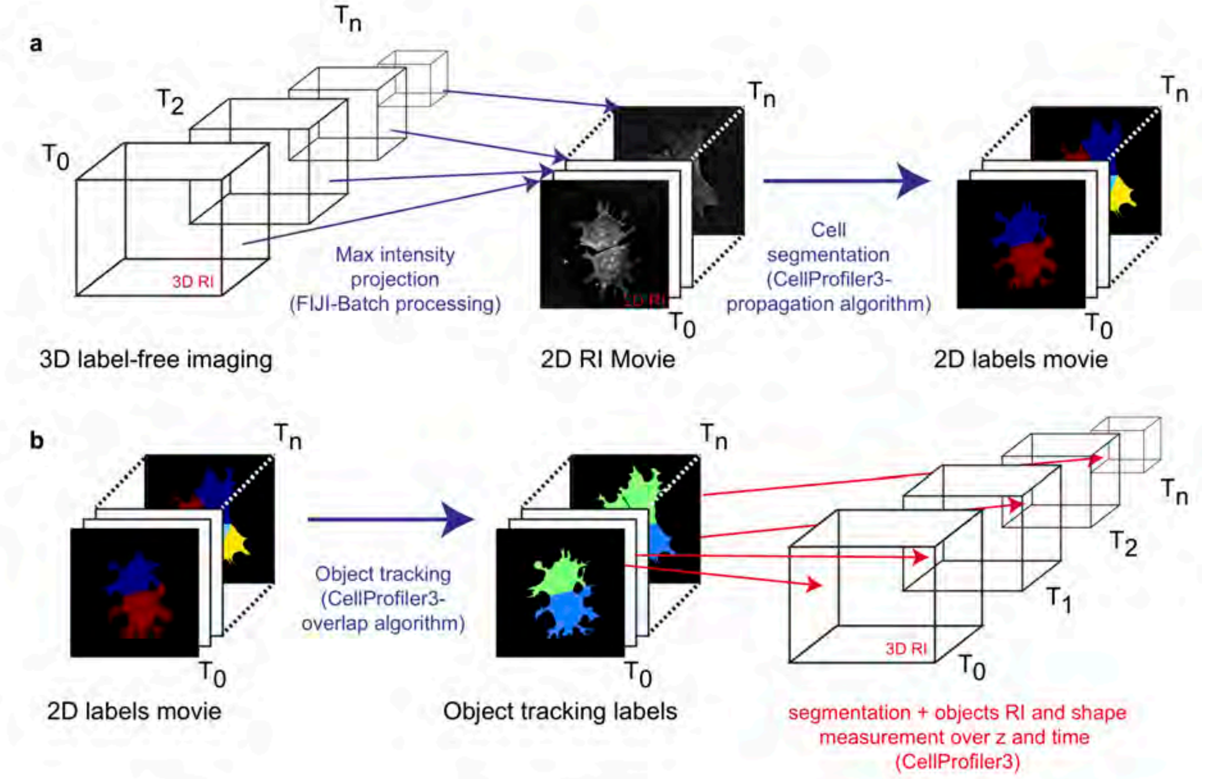

Figure 5 | Analysis of mESCs dry mass dynamics over cell division: computational analysis workflow. a, 3D Refractive index (RI) maps collected over an entire cell cycle are projected as 2D RI maps. Entire cells are detected in each frame using the propagation algorithm of Cellprofiler3 (CP3). **b**, Cell tracking and measurement over z and time dimension in CP3. Masks are adapted at each z slice using the threshold defined during the cell detection in **a**. (related to S4 movie).

We could extract the volume and RI map of each cell object contained in time lapse experiment and perform object tracking with CP3 to be able to analyze the cell's features over time (Fig 6A, S4 movie). The details of the segmentation and tracking procedure show that the image analysis process is able to catch even fine details of cellular protrusions, separate objects properly and keep their identity with satisfying precision over time (Fig 6A and B). Quantifications show that the cells have a similar volume evolution until cytokinesis, and such trends are easily observable in S1 movie. Their volume increases steadily before mitosis to reach app. 6000 µm^3, it then quickly reaches app. 1500 µm^3 before daughter cells separate and spread out again. The mean dry mass, calculated from the RI based on a linear calibration model (31), increases during the rounding of the cell from app. 25x10-2 pg.µm^{-3} to 6x10-2 pg.µm^{-3} before going back to 25x10-2 pg.µm^{-3}. More surprisingly, the total dry mass of cells decreases during mitosis, since we are confident in the precision of our segmentation, this observation can be interpreted in two ways, *i)* a significant amount of cellular material is excluded from the cell during the mitotic roundup, in direct contradiction with recent data showing that cell mass accumulates also during mitosis (45), this observation could be specific to the observed mESCs or *ii)* the linear calibration model (31) linking RI and dry mass does not apply during mitosis due to dry mass reorganization(46) or even phase transition.

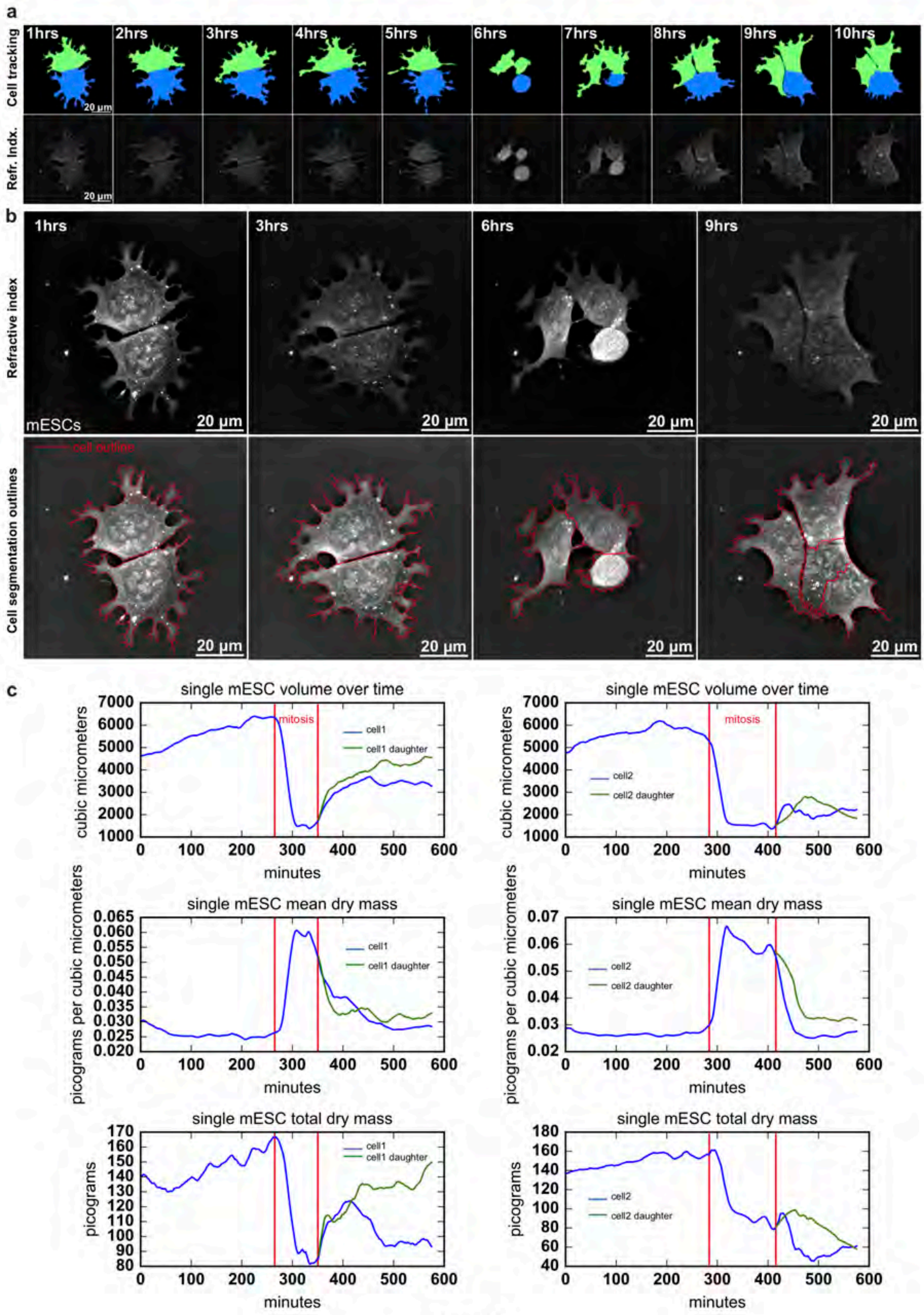

Figure 6 | mESCs dry mass dynamics over cell division suggests significant dry mass loss during mitosis. a, Time series of mouse embryonic stem cells (mESCs) refractive index (RI) maps and tracking masks obtained with Cellprofiler3 (CP3). Green and blue signify the lineage. **b**, Close up view on 1, 3, 6 and 9 hours mESCs RI maps and related segmentations overlays details. **c**, 10 hours long time traces of *(row1)* single mESC volume (*row2*) single

mESC mean dry mass and (*row3*) single mESC total dry mass for (*column1*) mESC 1 or (*column2*) mESC 2 and their daughters. (related to S1 and S4 movie).

Analysis of nuclear rotation using feature-matching and homography

Our unique capacity to observe multiple cellular organelles all at once within mouse embryonic stem cells (mESCs), and this over long time periods allowed us to capture a phenomenon of organelle spinning that occurs before mitosis. Rotations around an axis perpendicular to the substratum were observed for the first time in the 50's in epithelial cells, nerve cells(47) or HeLa cells(48) and was largely ignored until the mid-80's where the concept of nuclear rotation *per se* was challenged, with two proposed phenomenon explaining the observations made at that point in time. The first model was based on a nucleoli displacement combined with karyoplasm streaming(49) where only the intra-nuclear material would rotate and not the nuclear membrane, the second model supported a full nuclear rotation helped by the cytoskeleton(50,51) and with a major role of dynein in the process(52). We observe here in mESCs that such cellular event involves at least the entire nucleus, including the nuclear membrane and nucleoli that conserve their shape, as well as lipid droplets, which supports the model of a rotation of the entire nuclei(50) and extends it with a concomitant cytosolic streaming revealed by lipid droplet rotation (Fig 7A, S5 movie). To pave the road towards a better understanding of such rotational phenomenon or of milder nuclear movement such as those seen in migration associated nuclear alignments(53,54) we developed a computer-vision approach in python to help quantify the characteristics of the observed rotations(Fig 7B). This method is based on the use of scale-invariant features transform (SIFT) descriptors(55) originally developed for matching different views of an object or of a scene. The SIFT feature matching was done between the 2D RI maps at T and T+1 from a time lapse experiment displaying cells with rotating organelles (Fig 7C). Those matching features were then used to define a fitting homography matrix describing the transformation of T into T+1 (Fig 7D). To do so we used the Random Sample Consensus (RANSAC) method(56–58). We finally extracted the rotation angle from the homography matrices and represented them in linear and rose plots in order to observe the rotational features of the phenomenon (Fig 7E and F). Our quantifications suggest that the phenomenon can vary greatly in time, from 10 hours to 45 minutes, but will only be stopped by mitosis. It varies also greatly in rotation magnitude, from 80 to 700 degrees, demonstrating that our approach can catch subtle nuclear movements as slow at 0.2 degree.min^{-1} or relatively quick rotations as fast as 13 degrees.min^{-1} (S10 Fig). It is common to observe pauses and bursts in rotations, with a homogeneity in rotation speed. In fact, our dataset does not display oscillations in the rotational behavior, we observed only one direction reversal on the most limited rotation we recorded. Nuclear rotations have recently been shown to be involved in the dormant state of mouse oocytes(59) or to be key in migration of fibroblasts(53), in this context, we expect our approach to be a useful tool for characterizing the molecular and cellular details of this organellar rotation with at its center a full nuclear rotation.

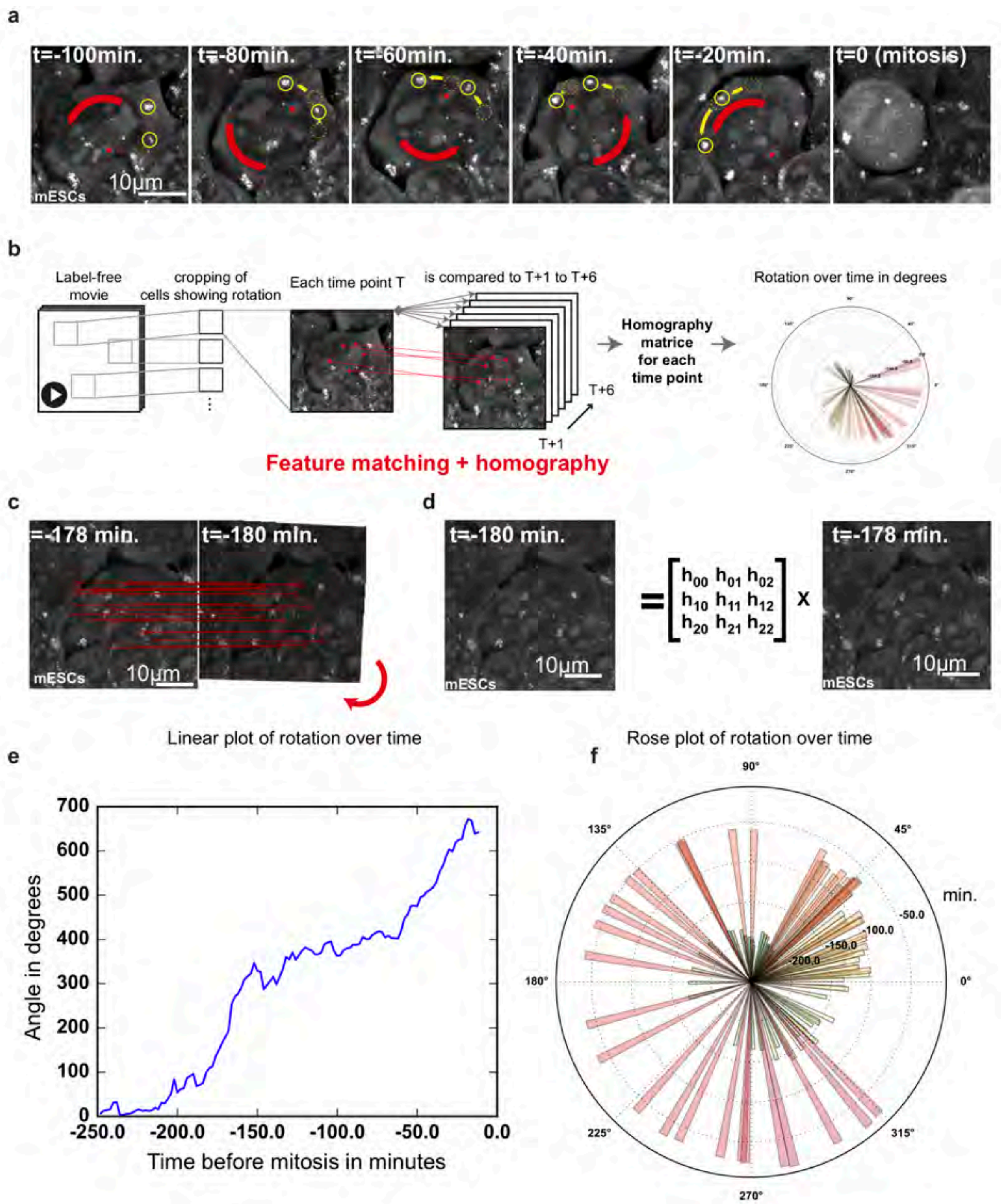

Figure 7 | Quantification of pre-mitotic intracellular rotation using features matching and homography. a, Time series of mouse embryonic stem cells refractive index (RI) maps showing nuclear rotation (Red Arrow) and lipid droplet rotation (Yellow Arrow and circles) before mitosis. **b,** Computational workflow for refractive index image processing and quantification of rotation over time. **c**, **d**, Homography matrices match resembling images subjected to translations and rotations. **e**, **f**, Plot and rose plot of intracellular rotations over time show the direction, extent, accelerations and pauses of the observed rotation. (related to S5 movie).

The previous section is titled "Results and Discussion"

Discussion

In this work we demonstrate that state-of-the-art holotomographic microscopy is suitable for the study cellular organelles. Its label-free nature combined to its excellent resolution, contrast and low phototoxicity allows to image specific organelles such as lipid droplets, mitochondria and pathologically lipid-loaded structures over long periods of time with great details and without sacrificing high temporal resolution. Importantly HTM has an inherent multiplexing capacity and allows to observe complex cellular dynamics such as organellar rotations that implicates many subcellular structures. The true limitation of HTM lies however in its main strength: because it works label-free, the produced images are extremely rich and crowded with information that the researcher must sort out in an exploitable way. To this extent we developed a set of image analysis approaches that are adapted for typical HTM label-free images and are easily reusable as they rely on existing and free image analysis software as well as on simple python code. With such resources, we want to demonstrate that label-free holotomography is ready for integrating quantitative biology research strategies and that it can provide new insights in a broad range of biological dynamics. Our quantitative study of lipid droplets (LD) identified insightful dynamics for understanding the process of early LD formation, we observe that newly forming LDs, all budding at the ER(29) are created at the expense of older LD's material, this observation implies that LDs must exchange material over relatively short time scales, and the ER offers the perfect media for that purpose(35). We also observe bursts in dry mass fluxes within LDs, suggesting a synchronization of fluxes in at least a subset of them, which could also be greatly facilitated by a continuity in their membrane through the ER. These observations are in support of a fluid and continuous connectivity between LDs (33–35). Perturbing the link between LD and ER (60) while doing similar time lapse experiments and quantifications of lipid droplets (LD) growth would provide new details on the connectivity existing between LDs and nascent ones or between growing LDs. More generally, the combination of our approach with genetic and chemical perturbations of the various machineries(29,35) identified for controlling LD maintenance and biogenesis could greatly improve our understanding of lipid droplets.

We applied a similar approach to record fine dynamic features of the late endosomal accumulation of lipid and cholesterol. This phenomenon was triggered with the compound U18666A that is classically used to emulate Nieman-Pick Type C phenotype(38). Our investigation of dry mass fluxes in particular extend our knowledge of the effect of U18666A. We observe that this compound triggers an accumulation of material in late endosomal structures by shifting the equilibrium between recycling and uptake. We observe that recycling still occurs since the average dry mass flux per object oscillates over time and regularly takes a negative value but not to such extent that it could counterbalance accumulation. We demonstrate that our simple label-free approach combining HTM to proper image analysis gives access to unique features such as vesicle shape, size, number and accumulation speed, or dry mass amounts and fluxes. Importantly this can be performed with minimal sample preparation, removing labeling- and phototoxicity-induced perturbations to which lipidic structures are very sensitive to(61,62). We believe that our work addresses a need for relevant, quantitative readouts that could accelerate the discovery of genes implicated in lysosomal storage diseases or the identification of drugs able to fight them(63).

We also developed a set of image analysis procedures that allow to segment and follow entire cells and applied them to track mouse embryonic stem cells before, during and after mitosis. This task is a technical challenge (39,40) that requires specific devices and methods(41–44) which led to contradicting results regarding what happens to cell size during mitosis (41,43,44). We demonstrate here that HTM images provide a sufficient level of details for performing very fine cellular

segmentation and tracking using CellProfiler3, which opens new possibilities for studying entire cell's dynamics.

We finally observed striking organellar rotations within mouse embryonic stem cells as well as in pre-adipocytes. Quantifying them was a challenge as to the best of our knowledge no preexisting tool in image analysis software would propose an integrated way to measure a coordinated multi-object rotation. We adapted a computer vision strategy developed for the matching of different views of macro-objects(55) in order to detect and match rotating objects(56–58) in HTM time lapse experiments. Such approach has the advantage to work without prior object segmentation and is therefore well adapted to the treatment of HTM images that show complex textures composed of multiple biological objects. This simple strategy allowed us to contribute significantly to the understanding of the phenomenon of organellar rotation. Firstly we could conclude that the entire nucleus rotates and not only the karyoplasm(49). Furthermore, we could show that such rotation extends to the cellular space as we also observe a rotation of lipid droplets, which could possibly be the consequence of a broader cytoplasmic streaming. Secondly, we characterized dynamic features of unperturbed rotations, observing those features such as speed, acceleration, rotation length and time with specific perturbations(50–52) will allow a better understanding of the molecular origin of the phenomenon and hopefully help highlighting its role for the cell's physiology and more particularly in fundamental functions such as the dormant state of germ cells(59).

Methods

Cell cultures and seeding. Cells were cultured in MEM medium complemented with 10% FBS, 1% Pen/Strep, 1% L-glutamine and 1% non-essential amino acids. Cells were seeded for 24 h at low concentration on glass bottom Fluorodishes of 25mm and 0.17mm thickness (World Precision Instrument Inc.). For mouse embryonic stem cells, Fluorodishes were first coated with Vitronectin following the manufacturer's protocol.

Transfection. Cells were retrieved with trypsin from tissue culture dishes and seeded in Fluorodishes. After 24 h, the medium was changed, and the cells were transfected using Fugene (Promega) according to the manufacturer's protocol.

Cell fixation. Fixation was performed with paraformaldehyde (PFA) or cold methanol. For PFA fixation, cells were first washed 3x with PBS. Then, 2mL of PFA (3%) was added for 30 minutes at 37°C. Afterwards, dishes were washed 3x with PBS. Quenching of the preparations was performed with 50 mM NH_4Cl in PBS at room temperature for 10 minutes before 3x PBS washes. Permeabilization was performed using 0.1% Triton X100 for 5 min at room temperature. In the case of methanol fixation, cells were washed 3x with PBS before adding pre-cooled methanol at -20°C for 4 min. Cells were washed 3x with PBS after fixation.

Immunofluorescence. Cells were seeded in Fluorodishes for 48 h (if so, including transfection) prior to fixation and permeabilization. Overnight blocking was performed in PBS with 0.5% BSA. Primary and secondary antibodies were applied for 30 minutes at room temperature each with in between 3x washes of PBS-0.5% BSA for 5 min. Finally, the preparation was washed again three times with PBS-0.5% BSA and post-fixed for 15 min at room temperature with 3% PFA followed by 3x PBS washes. Hoechst (Invitrogen) was used at 2µg/mL for 30 minutes at room temperature, BODIPY (Invitrogen) at 1µg/mL for 30 minutes in physiological conditions. Filipin (Sigma) was used at a dilution of 1:50 (from stock 50µg/ml).

Drug treatments. U18666A was applied to the cells at a dilution of 1:2000 (from a stock of 10 mg/ml). Oleic acid was applied at a dilution of 1:5000 (from stock 1mg/mL).

Imaging. Holo-tomographic Microscopy (HTM) in correlation with epifluorescence was performed on the 3D Cell-Explorer Fluo (Nanolive) using a 60x air objective (NA = 0.8) at a wavelength of λ=520nm (Class1 low power laser, sample exposure 0.2 mW/mm^2) and USB 3.0 CMOS Sony IMX174 sensor / Quantum E ciency (typical) 70 % (at 545 nm) / Dark Noise (typical) 6,6 e- / Dynamic Range (typical) 73,7 dB. The correlative acquisitions with bright-field, phase contrast and DIC were done on an Axiovert 200M (Zeiss) using a 63x objective (NA 1.4).

Live cell imaging. Physiological conditions for live cell imaging were reached with a top-stage incubator from oko-lab. A constant temperature of 37°C, an air humidity saturation as well as a level of 5% CO_2 was achieved throughout the acquisitions.

Image analysis of lipid droplets dynamics. An export was performed within the software STEVE that controls the HTM microscope to transform refractive index (RI) volumes into .tiff format. By doing so RI volumes can be read by the software FIJI. The exported 3D tiffs must be in float format to keep the explicit RI for each voxel value. The 3D RI volumes in .tiff format were then processed in batch within FIJI for performance purposes. 3D RI volumes were transformed into 2D RI maps using maximum intensity projections and were saved also as .tiff files. The resulting series of 2D frames could then be processed using CellProfiler3 (CP3) that do not support full 3D data analysis yet. The CellProfiler3 pipeline was designed to load each 2D RI map, segment the contained objects using the primary objects detection module and extract area, shape and intensity features using the measurements modules. A critical point for proper object detection was to use a manual threshold value. While automatic threshold detection is suited for most fluorescent microscopy images whose signal dynamics are related to a plethora of factors, RI images are quantitative and depends only on the nature of the biological object that is observed. Therefore, the threshold can be entered as a fixed

value, in our case 1.354, to ensure a relative stability of this first step of the detection procedure. The object size limits that we entered were designed to encompass the full spectrum of potential lipid droplet diameters from 1 to 7 pixels. The segmented objects were finally used to extract the area and the mean RI value of each LD in each frame of the time lapse experiment. The data was exported as a .csv file into a python environment where we used the extracted diameter of each lipid droplet to calculate its spherical volume, we could then calculate each LD dry mass content using a well-established linear calibration model(31). The data was finally plotted with the python library matplotlib.

Image analysis of late endosomal accumulation of cholesterol and lipids. An export was performed within the software STEVE that controls the HTM microscope to transform refractive index (RI) volumes into .tiff format. By doing so RI volumes can be read by the software FIJI. The exported 3D tiffs must be in float format to keep the explicit RI for each voxel value. The 3D RI volumes in .tiff format were then processed in batch within FIJI for performance purposes. 3D RI volumes were transformed into 2D RI maps using maximum intensity projections and were saved also as .tiff files. The resulting series of 2D frames could then be processed using CellProfiler3 (CP3) that do not support full 3D data analysis yet. The CellProfiler3 pipeline was designed to load each 2D RI map, segment the contained objects using the primary objects detection module and extract area, shape and intensity features using the measurements modules. A critical point for proper object detection was to use a manual threshold value. While automatic threshold detection is suited for most fluorescent microscopy images whose signal dynamics are related to a plethora of factors, RI images are quantitative and depends only on the nature of the biological object that is observed. Therefore, the threshold can be entered as a fixed value, in our case 1.347, to ensure a relative stability of this first step of the detection procedure. The object size limits were small and narrow from 1 to 3 pixels. The empirical process of finding the right values is fundamental to avoid detection of large clumps or the exclusion of unusually big structures. The segmented objects were finally used to extract the area and the mean RI value of each late endosomal structures in each frame of the time lapse experiment. The data was exported as a .csv file into a python environment where we used the extracted diameter to calculate its spherical volume, we could then calculate the dry mass content using a well-established linear calibration model (31). The data was finally plotted with the python library matplotlib.

Image analysis of mouse embryonic stem cells. An export was performed within the software STEVE that controls the HTM microscope to transform refractive index (RI) volumes into .tiff format. By doing so RI volumes can be read by the software FIJI. The exported 3D tiffs must be in float format to keep the explicit RI for each voxel value. The 3D RI volumes in .tiff format were then processed in batch within FIJI for performance purposes. 3D RI volumes were transformed into 2D RI maps using maximum intensity projections and were saved also as .tiff files. The resulting series of 2D frames could then be processed using CellProfiler3 (CP3) that do not support full 3D data analysis yet. The CellProfiler3 pipeline was designed to load each 2D RI map and to rescale them such that intracellular details do no perturb the global cellular detection, to do so, a simple rescaling from 0 to 1 of RI values between 1.32 and 1.34 was required. The subsequent segmentations of the cell objects were done using the secondary objects detection module based on primary objects defined *a priori*, using the manual Primary Object detection module. The area, shape and intensity features were then extracted using the related measurements modules on the unmodified RI maps. We preferred using a manual threshold value within the secondary object detection module together with the propagation algorithm for the best cellular segmentation. While automatic threshold detection is suited for most fluorescent microscopy images whose signal absolute values are related to a plethora of factors, RI images are quantitative and depends only on the nature of the biological object that is observed. Therefore, the threshold can be entered as a fixed value, since images were rescaled between 0 and 1, the manual threshold value we used is 0.89. The object size limits were between 80 and 250 pixels.

The empirical process of finding the right values is fundamental to avoid the detection of under- or over-segmented cells. The objects obtained from the 2D RI frames segmentation were then used in CP3 to measure the objects in each z-frames at each timepoint, excluding any voxel whose RI was out of the RI range of the objects' RI defined at the previous step. The data was exported as a .csv file into a python environment, the extracted area and RI values from each z-frame and time points where then reassembled to obtain 3D measurements from which we could for example calculate total and mean dry mass using a well-established linear calibration model (31). The data was finally plotted with the python library matplotlib.

Analysis of organellar rotations. An export was performed within the software STEVE that controls the HTM microscope to transform refractive index (RI) volumes into .tiff format. By doing so RI volumes can be read by the software FIJI. The exported 3D tiffs must be in float format to keep the explicit RI for each voxel value. The 3D RI volumes in .tiff format were then processed in batch within FIJI for performance purposes. 3D RI volumes were transformed into 2D RI maps using maximum intensity projections and were saved also as a .tiff files. The .tiff files opened as a stack in FIJI are then cropped to keep only one cell presenting rotating nuclei and organelles in the field of view. The resulting smaller 2D RI frames were then processed with a custom python algorithm implementing the feature matching and homography procedures of the OpenCV library. The first step consists in detecting at least 10 scale-invariant features transform (SIFT) descriptors(55) for at least each 2D RI frames adjacent pairs based on Lowe's ratio test. The identical features from T to T+1 were then used to define a fitting homography matrix describing the transformation of T into T+1. To do so we used the Random Sample Consensus (RANSAC) method (56–58). The rotation angle was then extracted from the homography matrices and plotted as regular and rose plots using the matplotlib python library. For the details and reuse of the procedure please find an adaptable python algorithm in the supplementary files.

Acknowledgment

We thank the LipidX consortium and the NCCR chemical biology for funding this work. We thank José Artacho from EPFL's Imaging Platform for his great help with the phase contrast and DIC correlative acquisitions. We thank Sebastien Equis, Lisa Polaro and Yann Cotte for precious redaction help and discussions. In addition, we thank Hubert Becker for hand-labelling of mitochondria and lipid droplets, Aleksandra Mandic for providing mESC, Kristina Shoonjans for the Hepa 1.6 cells and Pierre Gönczy for the U2OS cells, Sebastian Jessberger for KDEL-GFP, Anne-Laure Mahul for Mito-YFP, Tom Kirchhausen for Emerin-GFP and Jean Grunberg for anti-LBPA.

Data Availability

The data supporting this study are available from the corresponding authors upon reasonable request.

References

1. Cuche E, Marquet P, Depeursinge C. Simultaneous amplitude-contrast and quantitative phase-contrast microscopy by numerical reconstruction of Fresnel off-axis holograms. Appl Opt. 1999;38(34):6994–7001.
2. Gureyev TE, Roberts A, Nugent KA. Phase retrieval with the transport-of-intensity equation: matrix solution with use of Zernike polynomials. JOSA A. 1995;12(9):1932–1941.
3. Jena BP. Methods in nano cell biology. Vol. 90. Academic Press; 2008.
4. Bon P, Maucort G, Wattellier B, Monneret S. Quadriwave lateral shearing interferometry for quantitative phase microscopy of living cells. Opt Express. 2009;17(15):13080–13094.
5. Devaney AJ. A filtered backpropagation algorithm for diffraction tomography. Ultrason Imaging. 1982;4(4):336–350.
6. Zetie KP, Adams SF, Tocknell RM. How does a Mach-Zehnder interferometer work? Phys Educ [Internet]. 2000 Jan;35(1):46–8. Available from: http://stacks.iop.org/0031-

9120/35/i=1/a=308?key=crossref.fd2267a7959e18f7a165f773542c952b
7. Bao C, Barbastathis G, Ji H, Shen Z, Zhang Z. Coherence Retrieval Using Trace Regularization. SIAM J Imaging Sci. 2018;11(1):679–706.
8. Wolf E. Three-dimensional structure determination of semi-transparent objects from holographic data. Opt Commun. 1969;1(4):153–6.
9. Cotte Y, Toy MF, Pavillon N, Depeursinge C. Microscopy image resolution improvement by deconvolution of complex fields. Opt Express [Internet]. 2010 Sep 13;18(19):19462. Available from: https://www.osapublishing.org/oe/abstract.cfm?uri=oe-18-19-19462
10. Cotte Y, Toy F, Jourdain P, Pavillon N, Boss D, Magistretti P, et al. Marker-free phase nanoscopy. Nat Photonics. 2013;7(2):113–117.
11. Pollaro L, Equis S, Dalla Piazza B, Cotte Y. Stain-free 3D Nanoscopy of Living Cells. Opt Photonik. 2016;11(1):38–42.
12. Kim K, Lee S, Yoon J, Heo J, Choi C, Park Y. Three-dimensional label-free imaging and quantification of lipid droplets in live hepatocytes. Sci Rep. 2016;6.
13. Ali A, Abouleila Y, Amer S, Furushima R, Emara S, Equis S, et al. Quantitative live single-cell mass spectrometry with spatial evaluation by three-dimensional holographic and tomographic laser microscopy. Anal Sci. 2016;32(2):125–127.
14. Bennet M, Gur D, Yoon J, Park Y, Faivre D. A Bacteria-Based Remotely Tunable Photonic Device. Adv Opt Mater. 2017;5(1).
15. Dubois F, Yourassowsky C, Monnom O, Legros J-C, Debeir O, Van Ham P, et al. Digital holographic microscopy for the three-dimensional dynamic analysis of in vitro cancer cell migration. J Biomed Opt. 2006;11(5):054032–054032.
16. Choi W, Fang-Yen C, Badizadegan K, Oh S, Lue N, Dasari RR, et al. Tomographic phase microscopy. Nat Methods [Internet]. 2007;4(9):717–9. Available from: http://www.nature.com/doifinder/10.1038/nmeth1078
17. Schürmann M, Scholze J, Müller P, Guck J, Chan CJ. Cell nuclei have lower refractive index and mass density than cytoplasm. J Biophotonics. 2016;9(10):1068–76.
18. Maycock J, Hennelly BM, Mcdonald JB, Castro A, Naughton TJ. discrete Fourier filtering. 2007;24(6):1617–22.
19. Umin YWU, Heng HAC, En YOW, Hen XINC, Ang YIW. Coherent noise reduction of phase images in digital holographic microscopy based on the adaptive anisotropic diffusion. 2018;57(19):5364–70.
20. Schneider CA, Rasband WS, Eliceiri KW. NIH Image to ImageJ: 25 years of image analysis. Nat Methods [Internet]. 2012;9(7):671–5. Available from: http://www.nature.com/doifinder/10.1038/nmeth.2089
21. Carpenter AE, Jones TR, Lamprecht MR, Clarke C, Kang IH, Friman O, et al. CellProfiler: image analysis software for identifying and quantifying cell phenotypes. Genome Biol [Internet]. 2006 Jan [cited 2014 Jan 20];7(10):R100. Available from: http://www.pubmedcentral.nih.gov/articlerender.fcgi?artid=1794559&tool=pmcentrez&rendertype=abstract
22. Chum O, Matas J, Kittler J. Locally Optimized RANSAC. 2010;236–43.
23. Delponte E, Isgrò F, Odone F, Verri A. SVD-matching using SIFT features. Graph Models. 2006;68(5–6):415–31.
24. Zernike F. Phase contrast, a new method for the microscopic observation of transparent objects. Physica [Internet]. 1942 Jul;9(7):686–98. Available from: https://linkinghub.elsevier.com/retrieve/pii/S003189144280035X
25. Lang W. Nomarski Differential Interference Contrast Microscopy. ZEISS Inf. 1968;70:114–20.
26. James DI, Parone PA, Mattenberger Y, Martinou J-C. hFis1, a novel component of the mammalian mitochondrial fission machinery. J Biol Chem. 2003;278(38):36373–36379.
27. Jüngst C, Klein M, Zumbusch A. Long-term live cell microscopy studies of lipid droplet fusion dynamics in adipocytes. J Lipid Res [Internet]. 2013;54(12):3419–29. Available from: http://www.jlr.org/lookup/doi/10.1194/jlr.M042515
28. Folick A, Min W, Wang MC. Label-free imaging of lipid dynamics using Coherent Anti-stokes Raman Scattering (CARS) and Stimulated Raman Scattering (SRS) microscopy. Curr Opin Genet Dev [Internet]. 2011 Oct;21(5):585–90. Available from: http://linkinghub.elsevier.com/retrieve/pii/S0959437X11001389
29. Wilfling F, Haas JT, Walther TC, Jr RVF. Lipid droplet biogenesis. Curr Opin Cell Biol [Internet]. 2014 Aug;29(Figure 1):39–45. Available from: https://doi.org/10.1016/j.ceb.2014.03.008
30. Cohen B-C, Shamay A, Argov-Argaman N. Regulation of lipid droplet size in mammary epithelial cells by

remodeling of membrane lipid composition—A potential mechanism. PLoS One. 2015;10(3):e0121645.
31. Phillips KG, Jacques SL, McCarty OJT. Measurement of Single Cell Refractive Index, Dry Mass, Volume, and Density Using a Transillumination Microscope. Phys Rev Lett. 2012;109(11).
32. Long AP, Manneschmidt AK, Verbrugge B, Dortch MR, Minkin SC, Prater KE, et al. Lipid Droplet De Novo Formation and Fission Are Linked to the Cell Cycle in Fission Yeast. Traffic. 2012;13(5):705–14.
33. Kassan A, Herms A, Fernández-Vidal A, Bosch M, Schieber NL, Reddy BJN, et al. Acyl-CoA synthetase 3 promotes lipid droplet biogenesis in ER microdomains. J Cell Biol. 2013;203(6):985–1001.
34. Wilfling F, Wang H, Haas JT, Krahmer N, Gould TJ, Uchida A, et al. Triacylglycerol synthesis enzymes mediate lipid droplet growth by relocalizing from the ER to lipid droplets. Dev Cell. 2013;24(4):384–99.
35. Barneda D, Christian M. Lipid droplet growth: regulation of a dynamic organelle. Curr Opin Cell Biol [Internet]. 2017;47:9–15. Available from: http://dx.doi.org/10.1016/j.ceb.2017.02.002
36. Mishra R, Simonson MS. Saturated free fatty acids and apoptosis in microvascular mesangial cells: Palmitate activates pro-apoptotic signaling involving caspase 9 and mitochondrial release of endonuclease G. Cardiovasc Diabetol. 2005;4:1–12.
37. Kobayashi T, Beuchat M-H, Lindsay M, Frias S, Palmiter RD, Sakuraba H, et al. Late endosomal membranes rich in lysobisphosphatidic acid regulate cholesterol transport. Nat Cell Biol. 1999 Jun;1(2):113–8.
38. Cenedella RJ. Cholesterol synthesis inhibitor U18666A and the role of sterol metabolism and trafficking in numerous pathophysiological processes. Lipids. 2009;44(6):477–87.
39. Zangle TA, Teitell MA. Live-cell mass profiling: an emerging approach in quantitative biophysics. Nat Methods [Internet]. 2014 Dec 25;11(12):1221–8. Available from: http://www.nature.com/articles/nmeth.3175
40. Midtvedt D, Olsén E, Höök F, Jeffries GDM. Label-free spatio-temporal monitoring of cytosolic mass, osmolarity, and volume in living cells. Nat Commun [Internet]. 2019 Dec 21;10(1):340. Available from: http://dx.doi.org/10.1038/s41467-018-08207-5
41. Zlotek-Zlotkiewicz E, Monnier S, Cappello G, Le Berre M, Piel M. Optical volume and mass measurements show that mammalian cells swell during mitosis. J Cell Biol [Internet]. 2015 Nov 23;211(4):765–74. Available from: http://www.jcb.org/lookup/doi/10.1083/jcb.201505056
42. Son S, Kang JH, Oh S, Kirschner MW, Mitchison TJ, Manalis S. Resonant microchannel volume and mass measurements show that suspended cells swell during mitosis. J Cell Biol. 2015;211(4):757–63.
43. Boucrot E, Kirchhausen T. Mammalian cells change volume during mitosis. PLoS One. 2008;3(1):1–3.
44. Habela CW, Sontheimer H. Cytoplasmic Volume Condensation Is an Integral Part of Mitosis. Cell Cycle [Internet]. 2007 Jul 28;6(13):1613–20. Available from: http://www.tandfonline.com/doi/abs/10.4161/cc.6.13.4357
45. Miettinen TP, Kang JH, Yang LF, Manalis SR. Mammalian cell growth dynamics in mitosis. Elife. 2019;8:1–29.
46. Stewart MP, Helenius J, Toyoda Y, Ramanathan SP, Muller DJ, Hyman AA. Hydrostatic pressure and the actomyosin cortex drive mitotic cell rounding. Nature [Internet]. 2011;469(7329):226–31. Available from: http://dx.doi.org/10.1038/nature09642
47. Pomerat C m. Rotating nuclei in tissue cultures of adult human nasal mucosa. Exp Cell Res [Internet]. 1953;5(1):191–6. Available from: http://ac.els-cdn.com/0014482753901049/1-s2.0-0014482753901049-main.pdf?_tid=abf453f4-80d2-11e5-bdd3-00000aacb35d&acdnat=1446407982_aff03ad33162eaff02601979bc4afc75
48. Leone V, Hsu TC, Pomerat CM. Cytological studies on HeLa, a strain of human cervical carcinoma. Zeitschrift f�r Zellforsch und Mikroskopische Anat [Internet]. 1955;41(5):481–92. Available from: http://link.springer.com/10.1007/BF00345357
49. De Boni U, Mintz A. Curvilinear, three-dimensional motion of chromatin domains and nucleoli in neuronal interphase nuclei. Science (80-) [Internet]. 1986 Nov 14;234(4778):863–6. Available from: http://www.sciencemag.org/cgi/doi/10.1126/science.3775367
50. Paddock SW, Albrecht-Buehler G. Rigidity of the nucleus during nuclear rotation in 3T3 cells. Exp Cell Res. 1988;175(2):409–13.
51. Paddock SW, Albrecht-Buehler G. Distribution of microfilament bundles during rotation of the nucleus in 3T3 cells treated with monensin. Exp Cell Res [Internet]. 1986 Apr;163(2):525–38. Available from: https://linkinghub.elsevier.com/retrieve/pii/0014482786900832
52. Levy JR, Holzbaur ELF. Dynein drives nuclear rotation during forward progression of motile fibroblasts. J Cell Sci [Internet]. 2008 Oct 1;121(19):3187–95. Available from: http://jcs.biologists.org/cgi/doi/10.1242/jcs.033878

53. Maninová M, Iwanicki MP, Vomastek T. Emerging role for nuclear rotation and orientation in cell migration. Cell Adhes Migr. 2014;8(1):42–8.
54. Zhu R, Liu C, Gundersen GG. Nuclear positioning in migrating fibroblasts. Semin Cell Dev Biol [Internet]. 2018;82:41–50. Available from: http://dx.doi.org/10.1016/j.semcdb.2017.11.006
55. Lowe DG. Distinctive Image Features from Scale-Invariant Keypoints. Int J Comput Vis [Internet]. 2004 Nov;60(2):91–110. Available from: http://link.springer.com/10.1023/B:VISI.0000029664.99615.94
56. Bhattacharya P, Gavrilova M. Improving RANSAC feature matching with local topological information. Proc 2012 9th Int Symp Vor Diagrams Sci Eng ISVD 2012. 2012;17–23.
57. Wu F, Fang X. An improved RANSAC homography algorithm for feature based image mosaic. Proc 7th WSEAS Int Conf Signal Process Comput Geom Artif Vis [Internet]. 2007;202–7. Available from: http://www.wseas.us/e-library/conferences/2007athensel/papers/564-359.pdf
58. Fischler MA, Bolles RC. Random sample consensus: a paradigm for model fitting with applications to image analysis and automated cartography. Commun ACM [Internet]. 1981 Jun 1;24(6):381–95. Available from: http://portal.acm.org/citation.cfm?doid=358669.358692
59. Nagamatsu G, Shimamoto S, Hamazaki N, Nishimura Y, Hayashi K. Mechanical stress accompanied with nuclear rotation is involved in the dormant state of mouse oocytes. 2019;1–12.
60. Wilfling F, Thiam AR, Olarte M-J, Wang J, Beck R, Gould TJ, et al. Arf1/COPI machinery acts directly on lipid droplets and enables their connection to the ER for protein targeting. Elife. 2014;3:1–20.
61. Phototoxicity revisited. Nat Methods. 2018;15(10):751.
62. Daemen S, van Zandvoort MAMJ, Parekh SH, Hesselink MKC. Microscopy tools for the investigation of intracellular lipid storage and dynamics. Mol Metab [Internet]. 2016;5(3):153–63. Available from: http://dx.doi.org/10.1016/j.molmet.2015.12.005
63. Pugach EK, Feltes M, Kaufman RJ, Ory DS, Bang AG. High-content screen for modifiers of Niemann-Pick type C disease in patient cells. Hum Mol Genet. 2018;27(12):2101–12.
64. Bolte S, Cordelieres FP. A guided tour into subcellular colocalization analysis in light microscopy. J Microsc. 2006;224(3):213–232.
65. Legland D, Arganda-Carreras I, Andrey P. MorphoLibJ: integrated library and plugins for mathematical morphology with ImageJ. Bioinformatics. 2016;32(22):3532–3534.

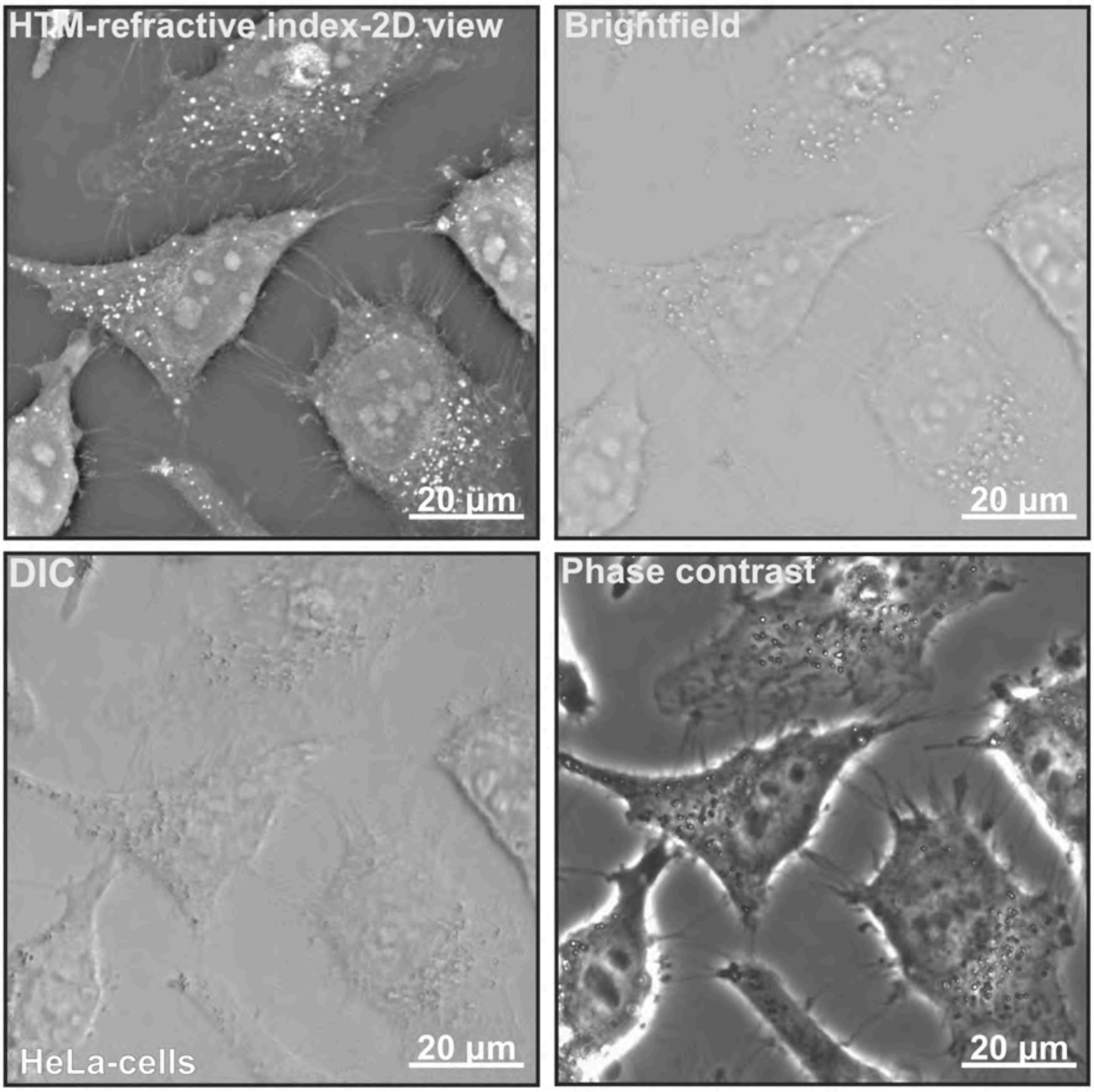

Extended data figure 1

Extended Data Figure 1 | Comparison of holotomographic microscopy and other label-free microscopy techniques. Brightfield, DIC and phase contrast images show limited resolution and major artifacts. On the contrary, HTM images show low background and resolution high enough to observe details such as filopodia or mitochondria.

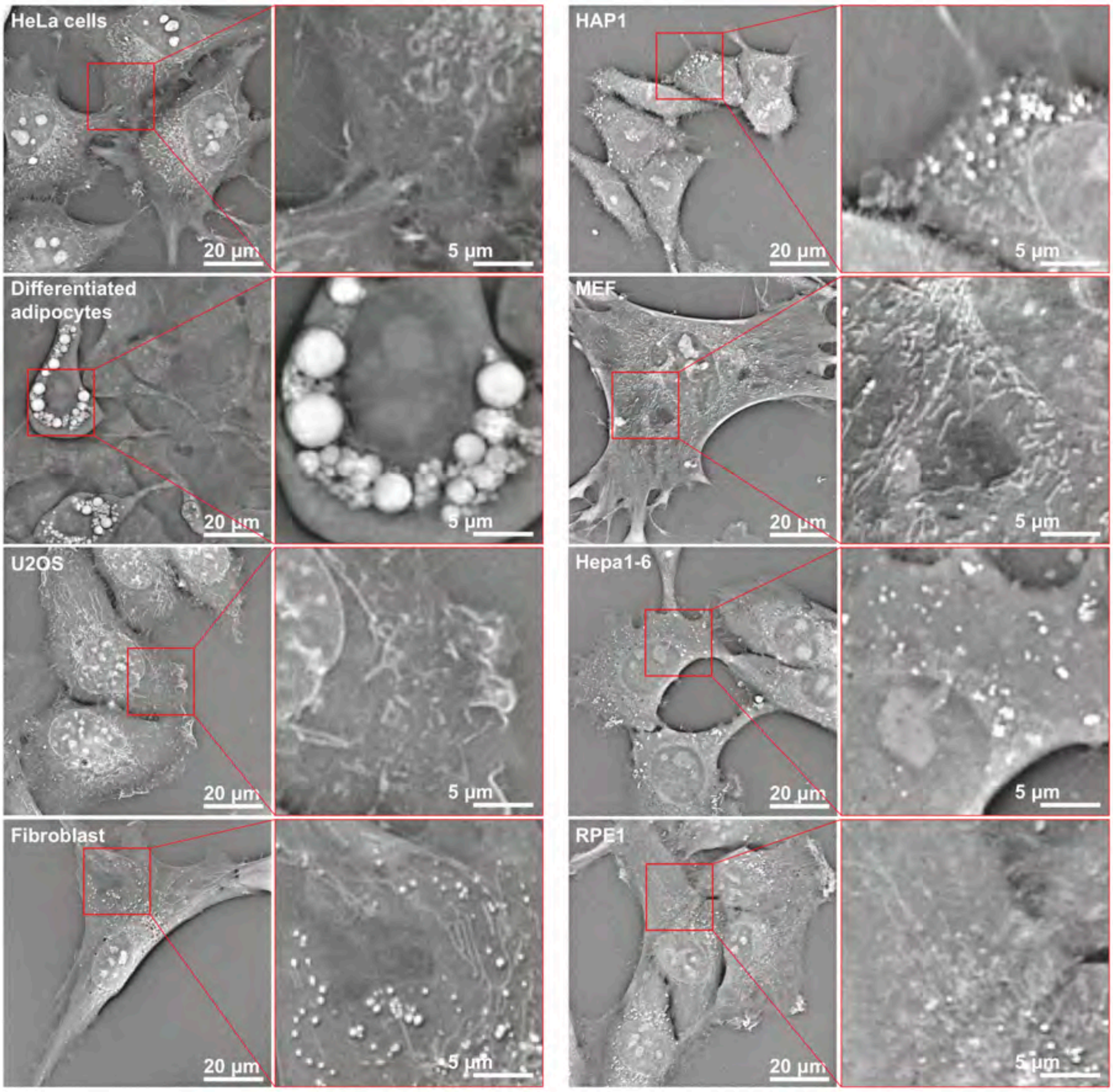

Extended data figure 2

Extended Data Figure 2 | Impact of methanol or paraformaldehyde fixation of HeLa cells on holotomographic microscopy results. Methanol fixation affect organelles structural integrity, paraformaldehyde is a viable solution for observing fixed cells with HTM.

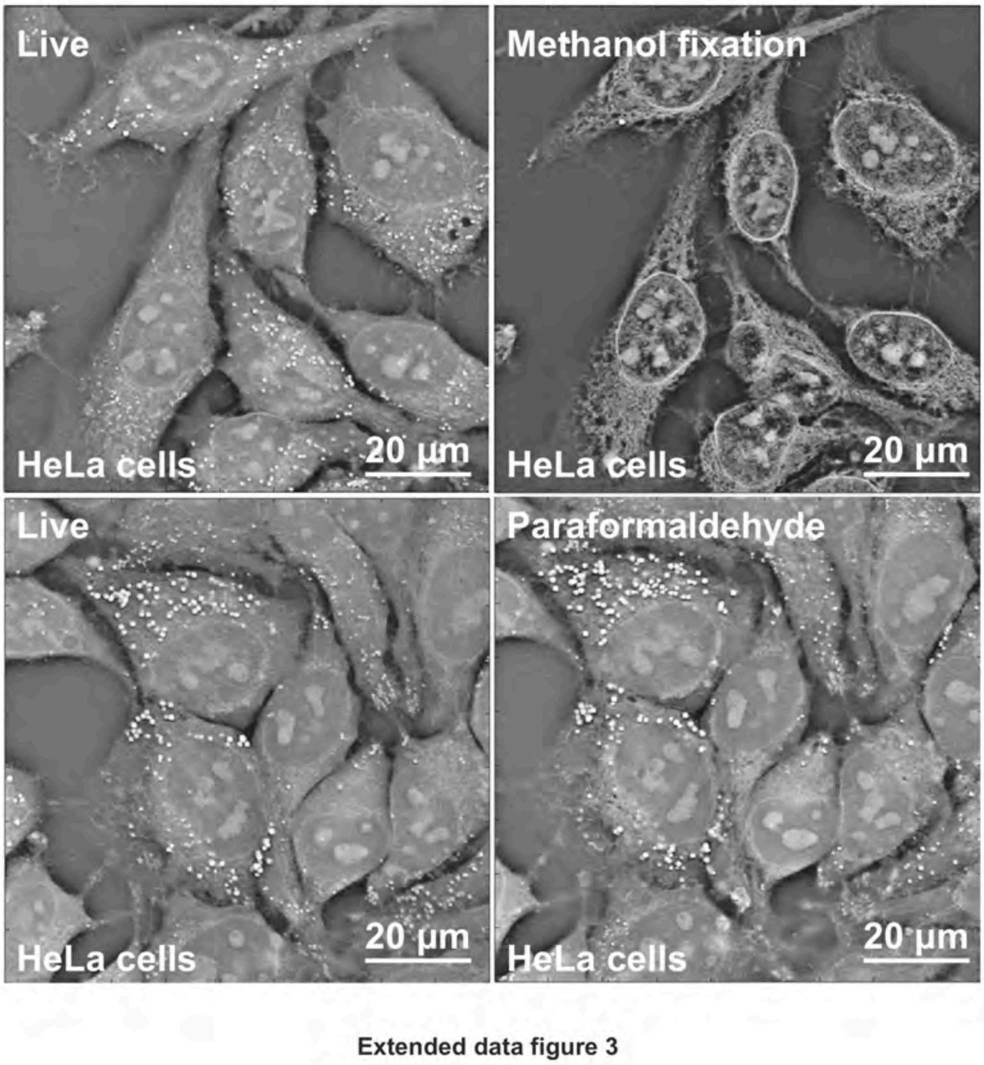

Extended Data Figure 3 | Aspect of various cell lines when observed with holotomographic microscopy. A wide range of cell lines are perfectly observable with holotomography.

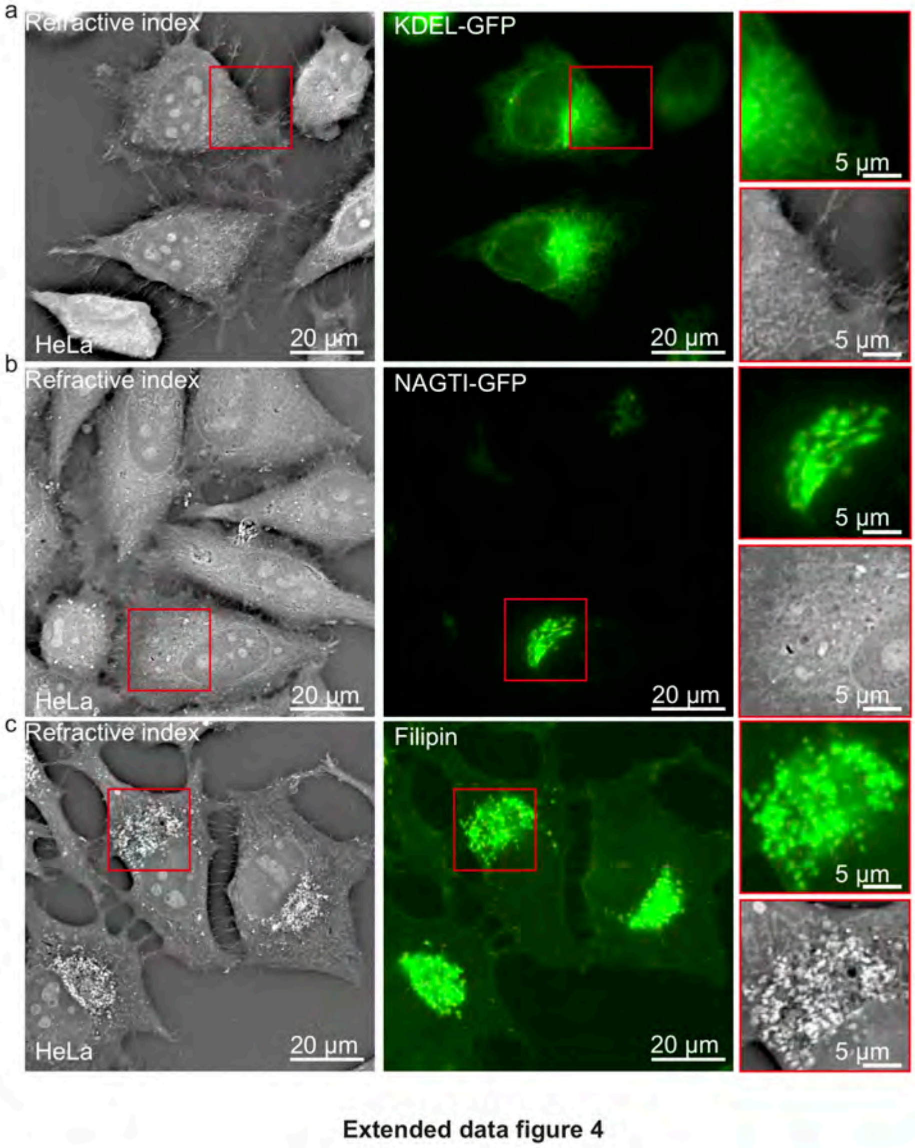

Extended Data Figure 4 | Comparison of specific fluorescent signals and refractive index map. Visual comparison of **a,** HeLa cells refractive index (RI) map to Golgi apparatus fluorescent signal (NAGTI-GFP) or **b,** to an endoplasmic reticulum fluorescent signal (KDEL-GFP) or **c,** to a late endosome accumulation signal (Filipin).

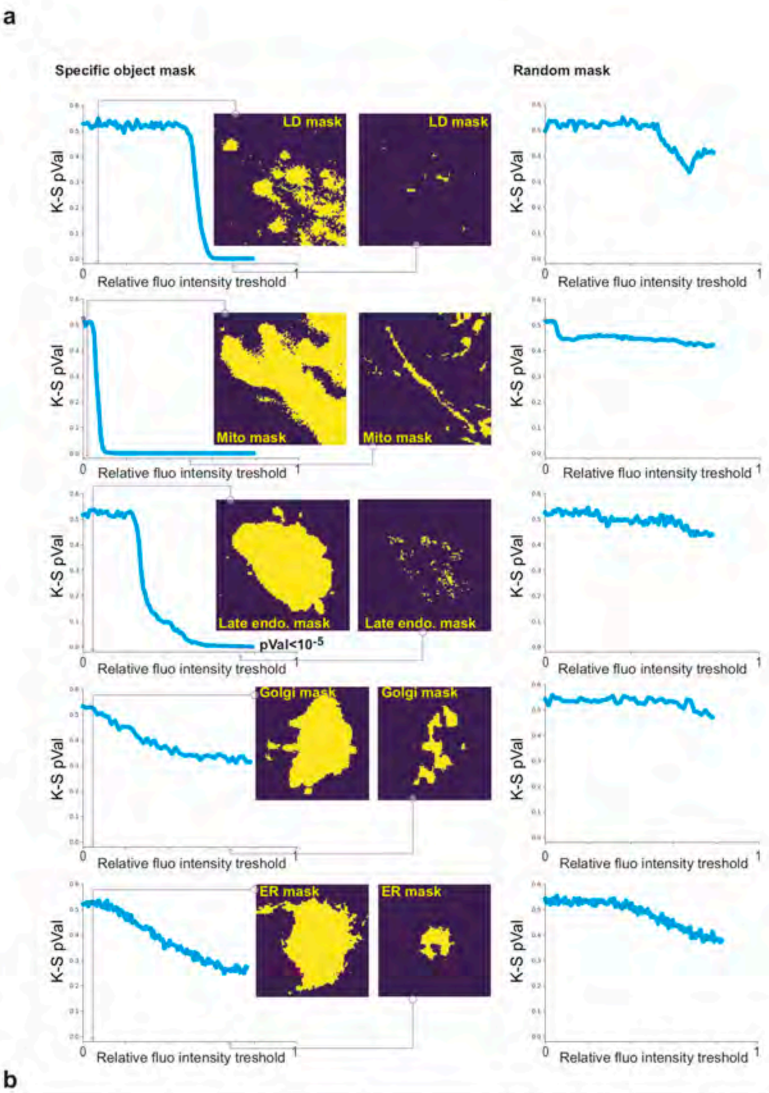

b

	Mitochondria	Lipid droplet	Late endosome	Endoplasmic reticulum	Golgi apparatus
Pearson correlation	0.64	0.59	0.4	~0	~0

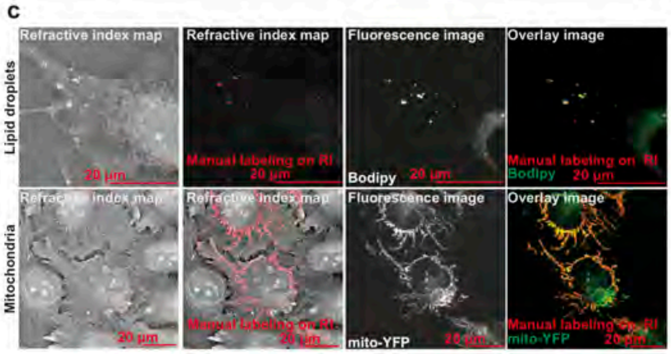

Extended data figure 5

Extended Data Figure 5 | Correlation of various fluorescent and RI signal using various metrics. a, Bootstrapped Kolmogorov-Smirnov test of the distribution of refractive index (RI) values under specific fluorescent signals against the global cellular RI distribution. Statistical test p-values are plotted as a function of the stringency of the threshold used to define the

fluorescence mask, from a specific or random fluorescent object signal. **b,** Pearson correlation of various fluorescent and RI signals.

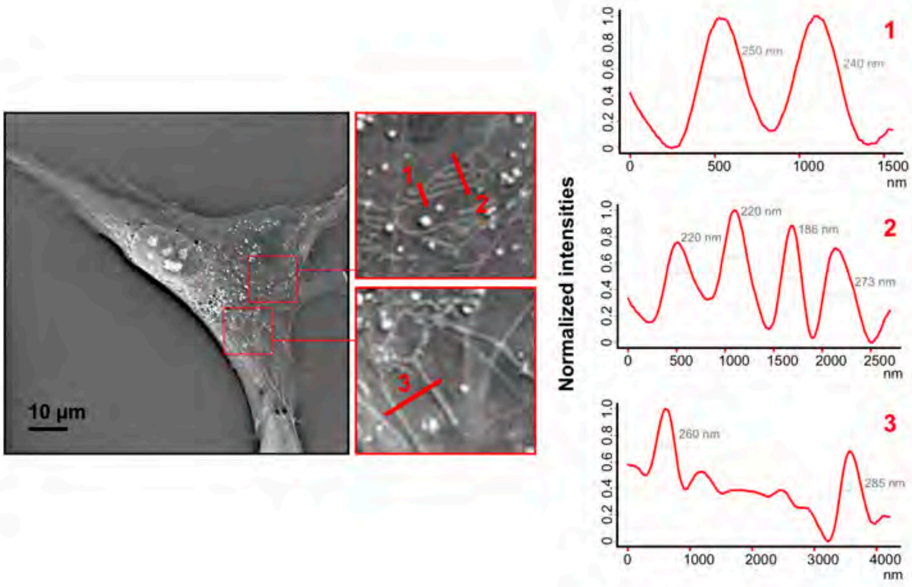

Extended data figure 6

Extended Data Figure 6 | Live imaging of mitochondrial fission and fusion events in mouse embryonic stem cells using holotomographic microscopy. Refractive index (RI) map of mouse embryonic stem cells times series showing mitochondrial fusion (blue arrow) and fission (yellow arrow) events. (related to S1 movie).

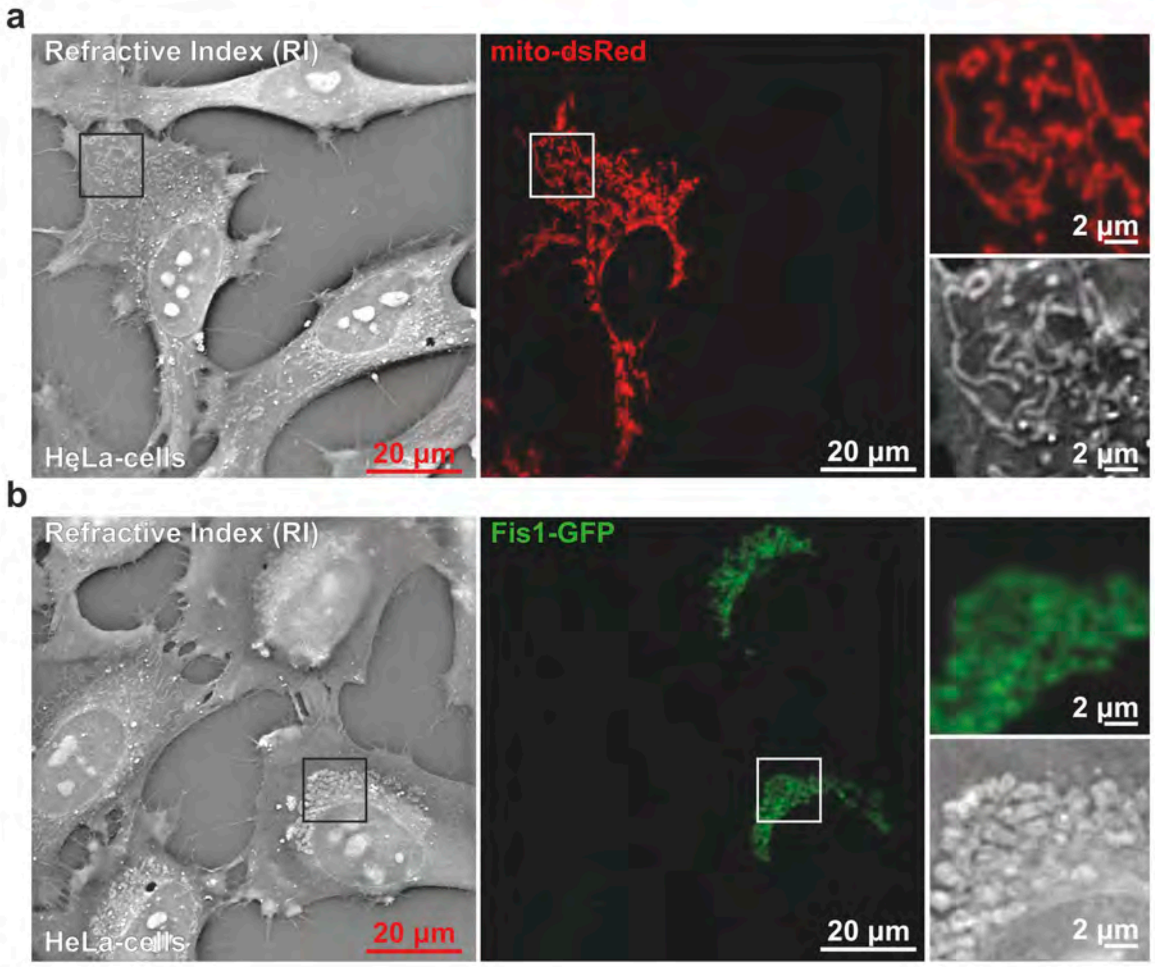

Extended data figure 7

Extended Data Figure 7 | Full width half maximum quantification of minimal mitochondrial thickness using FIJI software. The mitochondrial thickness has been measured within fibroblasts as the width observed at the half maximum of the mitochondrial signal distribution. The signal distributions along transversal lines have been defined within the FIJI software on zero padded image and are represented in the red enlargement squares (red lines 1-3).

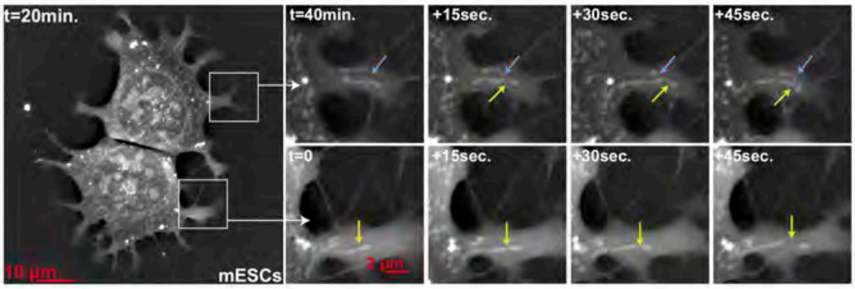

Extended data figure 8

Extended Data Figure 8 | Comparison of refractive index maps of cells when transfected with fluorescently tagged mitochondrial protein. Refractive index (RI) map of HeLa cells **a,** after transfection with a neutral mitochondrial Ds-Red marker or **b,** with the mitochondrial fusion protein Fis1-GFP.

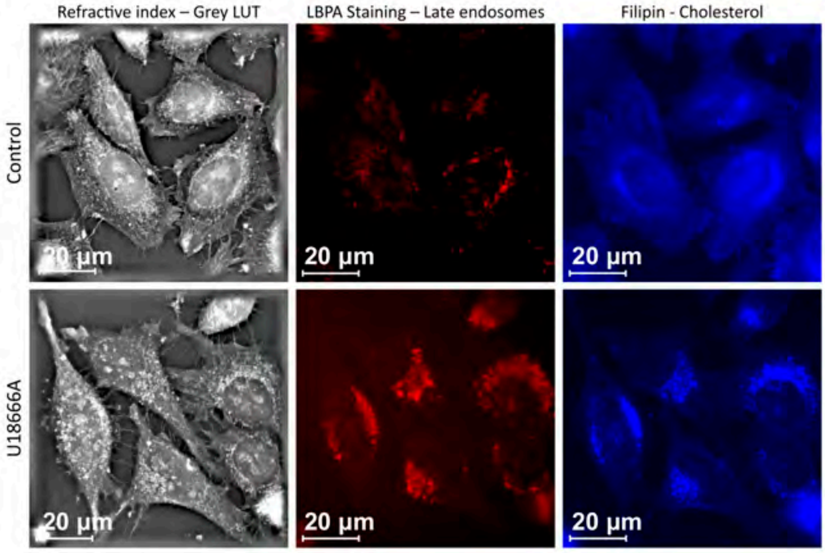

Extended data figure 9

Extended Data Figure 9 | Comparison between refractive index map and late endosome or cholesterol fluorescent signal. Compared to control, cells treated with U18666A show different perinuclear structures in the refractive index (RI) map that overlap with LBPA immunostaining and cholesterol filipin staining, indicating that the accumulation of cholesterol-rich late endosomes after treatment is observable in the RI map.

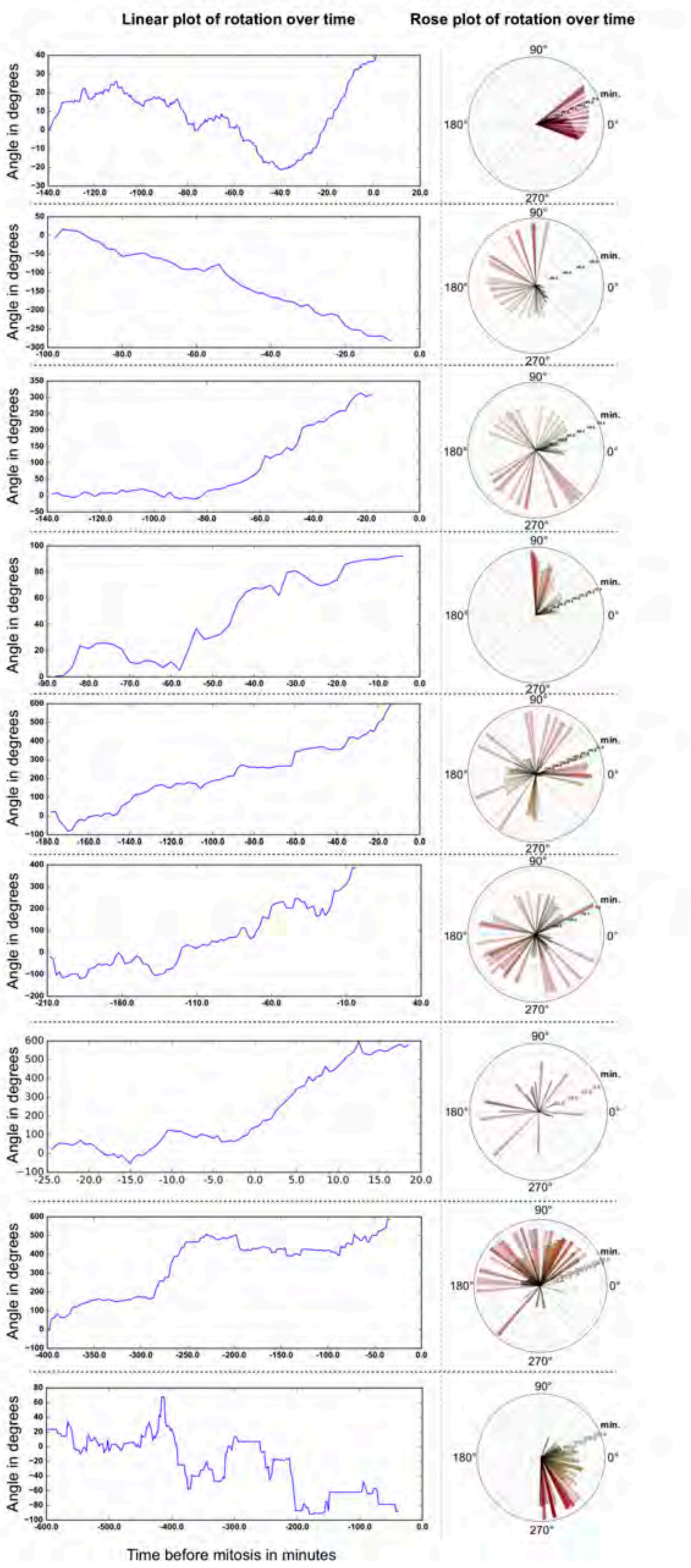

Extended Data Figure 10 | Quantification of pre-mitotic intracellular rotation using features matching and homography. Plot and rose plot of intracellular rotation over time show the direction, extent, accelerations and pauses of the observed rotation. (related to S5 movie).

Supplementary Video 1 | Live imaging of mouse embryonic stem cells using holotomographic microscopy. movie length, 10h, acquisition frequency, 4 image.min^{-1}. Related to Figure 6 and Extended Data Figure 6. This movie displays fine mitochondrial fusion and fissions events as well as a two full mitosis.

Supplementary Video 2 | Live imaging of lipid droplet growth in HeLa cells after oleic acid loading using holotomographic microscopy. movie length, 3h, acquisition frequency, 1 image.min^{-1}. Related to Figure 3. This movie displays lipid droplets growth that are quantified in the manuscript.

Supplementary Video 3 | Live imaging of late endosome accumulation in HeLa cells after U18666A treatment using holotomographic microscopy. movie length, 10h, acquisition frequency, 1 image.min^{-1}. Related to Figure 4. This movie displays the lipid accumulations in late endosomes that are quantified in the manuscript.

Supplementary Video 4 | Tracking segmentation labels of mouse embryonic stem cells Supplementary Video 1. movie length, 10h, acquisition frequency, 4 image.min^{-1}. Related to Figure 6.

Supplementary Video 5 | Live imaging of intra-cellular rotations in mouse embryonic stem cells and mouse pre-adipocyte using holotomographic microscopy. movie length is indicated in each sub movie square, acquisition frequency, 1 image.min^{-1}. Related to Figure 7 and Extended Data Figure 10.

python algorithm

Click here to access/download
Supporting Information
FeaturesMatchingPlusHomography.py
