# Supplementary material for: Image-based analysis of living mammalian cells using label-free 3D refractive index maps reveals new organelle dynamics and dry mass flux
Source: PLoS Biol. 2019 Dec 19;17(12):e3000553. doi: 10.1371/journal.pbio.3000553 (PMC6922317; doi:10.1371/journal.pbio.3000553)
Supplement: Attachment — Submitted filename: EditorialRequests-150919.docx [file pbio.3000553.s017.docx]

**Editorial requests:**

In particular, we will need you to provide a full and scholarly description of the working principle of the HTM technology, including a figure and a detailed description showing how the instrument is set up,

First of all, we would like to express our gratefulness for providing a constructive feedback and for allowing us to submit a new version of the manuscript. We provide in the introduction of the current manuscript a fully referenced description of the fundamental principles of HTM. Furthermore, we disclose all the details of the HTM setup used in the study, from the laser source to the camera we use, in particular we explain how micromirrors are key to the quality of our HTM images and provide all the necessary literature. We omitted no details of the microscopic device, both at the theoretical and technological levels.

and an explanation of how the absolute value of refractive index and index of refraction maps were obtained.

The manuscript provides state-of-the-art details and necessary references to understand how refractive index and index of refraction maps are obtained. Please note that fully developing the mathematics and algorithms behind such process is not possible in the format of the manuscript and has been extensively developed in previous reviews and publications, we provide all the necessary references for those interested in such information.

We would also need you to respond to all the other points raised by the reviewers with, wherever appropriate, changes to the text and the figures.

We provide a full point by point answer to reviewers’ comments in the submission files.

We would like you to provide a thorough study of lipid droplet biogenesis or the rotating nuclei that would justify the title of the article.

We believe that the current manuscript addresses this request for both the lipid droplet and organellar rotation topics. We greatly extended the quantification of lipid droplet growth and calculated dry mass fluxes within lipid droplets thanks to the fine segmentation of each lipid droplets and measurement of refractive index over time. This shines light on previously unknown dynamics and support the model of a fluid connectivity between existing and nascent lipid droplets through the ER membrane.

The phenomenon of organellar rotations that we report in this work is poorly known and likely very complex, in order to pave the road toward a better understanding of it, we expanded our data set and data analysis and developed a computer-vision approach in python to help quantify the characteristics of complex patterns motions in an unsupervised way, whether it is rotation in this case or any type of motion. This method is based on the use of scale-invariant features transform (SIFT) descriptors originally developed for matching different views of an object or of a scene. We then use homography matrices to calculate the rotation angles over time. Our quantifications suggest that the phenomenon can vary greatly in time, from 10 hours to 45 minutes, and in rotation magnitude, from 80 to 700 degrees, demonstrating that our approach can catch subtle nuclear movements as slow at 0.2 degree.min^-1^ or relatively quick rotations as fast as 13 degrees.min^-1^. Nuclear rotations have recently been shown to be involved in the dormant state of mouse oocytes or to be key in migration of fibroblast, in this context, we expect our approach and observations to be essential.

Finally, we will need you to provide full disclosure of all real or potentially perceived competing interests that you might have.

I have fully disclosed it in the related field during the submission process. I am an employee and a minor shareholder of Nanolive SA who produces the microscope used in the study.
