# Supplementary material for: Image-based analysis of living mammalian cells using label-free 3D refractive index maps reveals new organelle dynamics and dry mass flux
Source: PLoS Biol. 2019 Dec 19;17(12):e3000553. doi: 10.1371/journal.pbio.3000553 (PMC6922317; doi:10.1371/journal.pbio.3000553)
Supplement: Attachment — Submitted filename: 291019-Answers.docx [file pbio.3000553.s020.docx]

**Answers to reviewers comments:**

Rev. 1:

The revised manuscript is substantially improved. The authors have responded effectively to the points raised in the initial round of review, most notably by explaining the biological significance of the observed organelle rotation in mitotic cells. This is an excellent demonstration of the capabilities of the technique. The technique itself is described clearly and the discussion appropriately places the focus on the technique rather than the commercial implementation.

The present version of the manuscript is suitable for publication in PLOS Biology.

We would like to thank the Reviewer 1 for his fair and helpful comments and remarks. It greatly helped us. We are pleased to read that our work is suitable for publication and especially that our effort to focus on the science has been recognized.

Rev. 2:

This is a revised manuscript presenting a label-free 3D refractive index imaging method based on holo-tomographic microscopy (HTM). I had to admit that the quality of the images and videos are very impressive. The HTM images have really good resolution and contrast, which compare favorably to bright-field, DIC and phase contrast microscopes. The authors tried to respond to the editor and reviewers’ comments and made a major revision to the manuscript. However, there are still several concerns with the current manuscript.

We are grateful that Reviewer 2 invested much time and efforts in correcting our manuscript. We are glad to read that our improvements were appreciated, and we were eager to improve the manuscript further with the relevant changes.

1. A similar manuscript from the authors was posted on BioRxiv in 2018. However, the authors listed are not exactly the same as the current submitted manuscript. Please explain.
<https://www.biorxiv.org/content/biorxiv/early/2018/09/04/407239.full.pdf>

We appreciate that Reviewer 2 ask us this question and understand the necessity for clarity. PLOS biology, as Reviewer 2 might not be aware of, requires that we provide such justification. We are happy to reiterate our explanation here. As acknowledged by both Reviewers, the manuscript has been profoundly revised, and the relative contributions significantly changed. The new authors line reflects that evolution in agreement with all the concerned people. The respective roles of the authors have been described in the submission process and I assume are accessible to the Reviewers. Minor contributions, as it is common practice, have been acknowledged in the dedicated section of the manuscript.

2. Even with the additional details in the introduction, it is still not clear how the HTM system used in the current study was configured and how image reconstruction was performed. Figure 1a is not clear nor sufficient. The cited references are not up to date (e.g. the most recent reference in Ref. 1-10 are from 2013; many others were from 10-20 years ago).

We are grateful for these comments as it allows us to reinforce the fact that the reported information is a direct reflection of the state of the art of the discipline.

Regarding the following specific point of Reviewer 2: the most recent reference in Ref. 1-10 are from 2013; The reference 7 is from **2018**, therefore the most recent reference in Ref. 1-10 is from **2018**, **not** 2013. We discuss further the “up-to-date-ness” of our refences later in the text.

Regarding the following comment: “it is still not clear how the HTM system used in the current study was configured”

We deeply apologize if we fail at understanding what is unclear to Reviewer 2 about the HTM system configuration. In absence of specifics, we can only reiterate that the microscope configuration has been explained in its entire details, between main text and the methods section. Reviewer 1 asked us a similar question during the previous reviewing round, we believe that the present communication of Reviewer 1 acknowledges the fact that we fully addressed the point. (*note that specific information asked by Reviewer 2 at point 5 regarding hardware specifications have been gladly added*).

We would like to bring to Reviewer 2 attention that citation 11 (*now 12 in the current version*) details the used holotomographic setup extensively:

***Pollaro L, Equis S, Dalla Piazza B, et al. Stain-free 3D Nanoscopy of Living Cells. Opt Photonik 2016; 11: 38–42***

Setup figures are displayed there. We believe it is important to focus on novelty -how HTM could bring novel cell biology discoveries- and to credit rather than reproduce the content of other published works.

Regarding the following comment: “The cited references are not up to date”

We would like to reinsure Reviewer 2 about the references 1 to 10 (*1 to 11 of the current version, excluding citation 6, which has been added thanks to Reviewer 2 suggestions – see answer to point 6*).

They are to the best of our knowledge, the relevant ones. It is unclear to us why Reviewer 2 considers them not up to date, being “old” is certainly not disqualifying. We made the specific effort of citing the seminal publications, those can be from as old as 1969, (Wolf E. Three-dimensional structure determination of semi-transparent objects from holographic data. Opt Commun. 1969;1(4):153–6) but are nonetheless the references that must be recognized.

Regarding the following comment: “it is still not clear […] how image reconstruction was performed”

We are really flattered to have triggered such an interest for the fundamentals of HTM. The topic of reconstruction has been extensively developed in previous reviews and publications and the present manuscript provides explanations on the topic that are, we believe, at the right level of details. We provide at the same time historical perspective by citing the seminal work of Wolf (*ref 8 – 9 in current version*) and two more recent publication (*ref.7 and 9 – 8 and 10 in current version*) that are the relevant entry points in the topic. The novelty of the paper does not reside in HTM itself and well-known reconstructions techniques, but in its application to cell biology. We believe that it is important to stay focused on bringing novelty and to not repeat concepts that have been extensively treated in publications and reviews.

3. If absolute values of the RI images were obtained, it would be important to show them in addition to the normalized images. A color bar can be added to the images to show the range of the absolute RI. It would be interesting to see the RI differences among various organelles, as well as variations between different cells.

We followed the suggestion of Reviewer 2 and we added a grayscale bar in all images to illustrate the scale of RI values.

4. No 3D views of the RI images were shown, although Figure 1b says so.

Figure 1B is a 3D view. We apologize regarding this very short answer, but it is in fact a 3D view. Representing the 3D aspect of an unlabelled flat cell on the 2D surface of a sheet is challenging but doable, as we can observe in figure 1B. We nonetheless improved the Figure legend of Figure 1B to explain the data further. Figure 2 contains also 3D representations of organelles whose “3D-ness” can be more quickly appreciated.

5. What is the depth range, axial resolution and temporal resolution of the HTM system? How small of a RI change can it detect?

We added this information in the methods section.

6. The manuscript can be further improved with its readability. For example, the introduction can explain the difference and compare the pros and cons of HTM and other quantitative phase imaging methods. Technical details of the system can go into the method section instead of introduction. Connections between different experiments can be better explained. Any biological insights from these experiments would be appreciated.

Regarding the following comment: “The manuscript can be further improved with its readability. For example, the introduction can explain the difference and compare the pros and cons of HTM and other quantitative phase imaging methods.” We profoundly understand the remarks of Reviewer 2 and recognize the fundamental interest in talking about HTM pros and cons. We should start by saying that pros and cons of HTM have been exposed in the present manuscript, and compared to another major label-free technique, Raman scattering, as it was very relevant regarding the biological topics we investigate. It would however totally transform the manuscript to discuss the pros and cons of HTM regarding other QPI methods, moreover, this has been done extensively in the past, and it actually took a full, wonderful review to tackle the topic:

***Park Y, Depeursinge C, Popescu G. Quantitative phase imaging in biomedicine. Nat Photonics. 2018;12(October).***

We felt also that we could add it to our reference list. (*now reference 6*)

We would like to say again that our goal is not to discuss HTM itself but to demonstrate how HTM can help cell biologists by providing concrete biological discoveries enabled by the combination of HTM and computer vision strategies. Again, we profoundly understand the remarks of Reviewer 2 and recognize the fundamental interest of it. However, an abundant literature exists already on the topic.

Regarding the remark “Connections between different experiments can be better explained” we can only apologize and admit that we fail in understanding Reviewer 2 point, if it concerns the overall progression of the paper, as a Resource and Methods format, our work is coherent in bringing *an image-based analysis of living mammalian cells imaged with HTM, that reveals new organelle dynamics and dry mass flux.* In the absence of specifics, we reiterate that we did our best to improve the text regarding this feeling of connection in the discussion of the manuscript.

Regarding the following comment: “Any biological insights from these experiments would be appreciated.” Assuming that we understood properly Reviewer 2’s comment, and that the terms “These experiments” concern all the experiments presented in the manuscript, we can with confidence say that it was the whole effort of the present manuscript to bring more biological insights. Reviewer 1 communication supports our idea that we succeeded in that aspect. In the absence of specific missing biological insights, we can only state again that the manuscript is providing new and impactful biological insights in Lipid droplet biology, trafficking, cell cycling and organelle dynamics, with concrete new knowledge and tools, all placed in their respective state-of-the-art.

7. There are also several mistakes/errors with uploaded figures and captions: Figures and captions for Extended Data Figure 2 and 3 are flipped; Caption of Extended Data Figure 7 refer to the Extended Data Figure 6. Caption for 8 points to Figure in 7; and caption for 6 points to Figure in 8; Extended Data Figure 5, what is shown in c?

We would like to thank Reviewer 2 for going in depth in the manuscript organization, those mistakes have all been fixed.
